# Warm‐wet transition restructures arid lake microbiomes and functional potential

**DOI:** 10.1002/imo2.70119

**Published:** 2026-07-08

**Authors:** Zhen Shen, Xingchen Li, Zhijun Gong, Yang Hu, Yongjiu Cai, Keqiang Shao, Guang Gao, Robbie M. Martin, Xiangming Tang

**Affiliations:** ^1^ State Key Laboratory of Lake and Watershed Science for Water Security, Nanjing Institute of Geography and Limnology Chinese Academy of Sciences Nanjing China; ^2^ University of Chinese Academy of Sciences Beijing China; ^3^ School of Earth and Environment Anhui University of Science & Technology Huainan China; ^4^ Department of Microbiology University of Tennessee Knoxville Tennessee USA

**Keywords:** arid lake microbiome, community assembly, cross domain network, functional redundancy, hydroclimatic shift, nitrogen cycling, warm‐wet transition

## Abstract

Over the past six decades, northwestern China has undergone a pronounced climatic transition from a “warm‐dry” to a “warm‐wet” regime, driving substantial shifts in lake water level (WL), salinity, and nutrient conditions. However, how this hydroclimatic transition restructures lake microbiomes and their functional potential remains poorly understood. Here, we used Lake Bosten, the largest inland lake in arid northwestern China, as a model ecosystem to examine the long‐term impacts of climate‐driven environmental change on microbial diversity, community assembly, network structure, and functional potential. We combined (1) meteorological and aquatic environmental records from 1958 to 2022, (2) decade‐separated field surveys of microbial communities (2010−2011 vs. 2021−2022) using amplicon sequencing and metagenomics, and (3) mesocosm experiments simulating projected future hydroclimatic conditions. Warm‐wet conditions influenced the Lake Bosten ecosystem through two main pathways: direct temperature effects and indirect WL mediated effects on hydrological connectivity and water chemistry. Field observations showed that the warm‐wet period was characterized by higher WL, lower salinity and total nitrogen, and increased bacterial and eukaryotic diversity. Community assembly shifted toward stronger homogeneous selection and weaker dispersal limitation in both bacterial and eukaryotic communities, consistent with enhanced hydrological connectivity. At the same time, both microbial domains showed reduced community stability, and their co‐occurrence networks exhibited lower complexity and structural stability. In contrast, broad metagenomic functional composition showed no significant shift despite pronounced taxonomic and network reorganization. Nitrogen cycling genes showed pathway‐specific redistribution, with enhanced nitrogen fixation and selected nitrification related genes but reduced nitrate reduction, denitrification, and dissimilatory nitrate reduction to ammonium (DNRA). Taxon‐function linkage further indicated that functional compensation was mainly mediated by replacement within bacterial functional guilds. Overall, our study links warm‐wet hydroclimatic change to altered water level, salinity, nutrient conditions, microbiome restructuring, cross domain network rewiring, and buffered functional potential in an arid lake.

## Highlights

- Warm‐wet transition restructured arid lake microbiomes and environmental conditions.
- Bacteria and eukaryotes showed shared but domain‐specific community responses.
- Within domain network complexity and structural stability declined under warm‐wet conditions.
- Functional redundancy and bacterial guild replacement buffered nitrogen cycling potential.

## Graphical abstract

This study summarizes how a long‐term warm‐dry to warm‐wet transition restructures microbiomes and functional potential in Lake Bosten, an arid lake in northwestern China. Warm‐wet hydroclimatic change increased water level and hydrological connectivity, while decreasing salinity and total nitrogen. These environmental shifts restructured both bacterial and eukaryotic communities, with reduced dispersal limitation and enhanced homogeneous selection. Bacterial diversity increased, whereas eukaryotic diversity showed a weaker response. Warm‐wet conditions weakened within domain community compositional stability and network structural stability, but strengthened bacteria‐eukaryote interactions. Despite pronounced taxonomic and network reorganization, broad functional potential was buffered through functional redundancy, cross domain coupling, and taxonomic replacement within bacterial functional guilds, particularly in nitrogen cycling pathways.

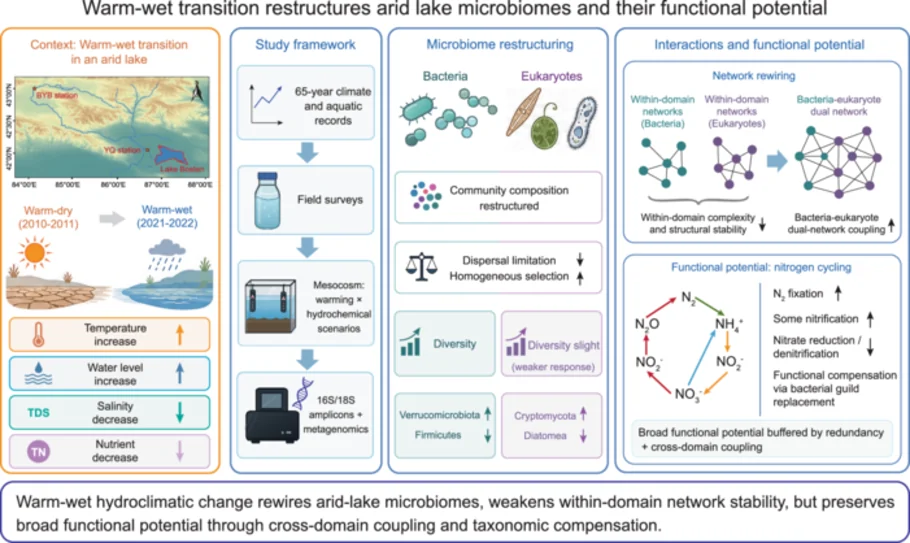

## 1 INTRODUCTION

Increasing atmospheric concentrations of greenhouse gases are intensifying global climate change [1], altering the intensity and distribution of temperature, evapotranspiration, and precipitation at both global and regional scales [2, 3, 4]. These shifts are reshaping dry and wet conditions, exerting far‐reaching impacts on water resources, water environments, and agricultural production [1, 5]. Generally, the paradigm “dry gets drier, wet gets wetter” is considered typical for global dry‐wet changes, and this pattern has been verified in marine ecosystems [3, 6]. However, studies have revealed that only about 10.8% of terrestrial areas conform to this paradigm, while approximately 9.5% show the opposite trend [3, 7]. Northwest China, located in the heart of the Eurasian continent, is a representative arid and semi‐arid region [3, 8, 9]. The unique geographical and climatic conditions in this region create an environment that is highly vulnerable and extremely sensitive to fluctuations between dry and wet climate regimes [10]. This sensitivity has profoundly impacted regional water resources, ecological security, and the risk of natural disasters, emerging as a critical concern for governments and the public alike [8, 9, 10]. Consequently, systematic research into the region's climatic changes and their ecological consequences holds significant scientific value and is crucial for informing strategies aimed at sustainable regional development.

Over the past few decades, research has revealed that between 1961 and 2020, the average temperature in Northwest China rose at a rate of 0.34°C/10a, exceeding the average rates for China (0.29°C/10a) and the global average (0.22°C/10a) [2]. Alongside temperature changes, precipitation in the region increased by 4.81 mm/year from 1959 to 2019 [10]. This increase in precipitation, coupled with rising lake water levels and runoff, indicates a climatic transition from a “warm‐dry” (WD) to a “warm‐wet” (WW) regime across Northwest China [3, 9]. However, asymmetric warming may enhance evapotranspiration rates, potentially offsetting the effects of increased precipitation and weakening the overall wetting trend [3]. These climatic shifts have introduced considerable uncertainty into regional water cycles and hydrological processes, particularly in arid‐zone lake systems [2, 3, 11]. Greater hydrological inputs have altered water chemistry, leading to pronounced fluctuations in total dissolved solids (TDS) and driving a dynamic cycle of salinization and desalinization [9, 12].

Lakes in arid and semi‐arid regions are highly sensitive to climate change, serving as valuable indicators of environmental evolution [9, 13, 14]. Researchers have conducted extensive studies on various aspects of these lakes, including sedimentary physical‐chemical properties [15, 16], changes in the number and area of lakes [17], water resources [18], water level fluctuations [19], and variations in water TDS [9]. Other research has examined lake salinization and vegetation degradation caused by changes in water resources and environments [20]. However, most of these studies have been conducted on a macro scale, focusing primarily on abiotic variables, such as water resources, changes in water quality, physical‐chemical properties, and the succession of vegetation. In contrast, relatively limited attention has been paid to biotic variables, particularly structural and functional diversity of lake ecosystems, in response to the combined effects of a warm‐wet climate.

Climate change significantly transforms the structure, function, and ecological processes of lake ecosystems across spatial and temporal scales [13, 21], leading to challenges such as reduced or enhanced ecosystem stability and shifts in steady states [22, 23]. Microbes, though a subset of ecosystem components, play essential roles in food webs, making their diversity and functional dynamics sensitive indicators of ecosystem change linked to climate change [24]. Recent studies indicate that warming reduces microbial diversity, ecological network complexity, and stability, primarily through environmental filtering, where certain species are either selected or eliminated due to shifts in environmental conditions, as well as altered biological interactions [25]. Precipitation, on the other hand, indirectly enhances microbial diversity and alters algal community structures by diluting salinity and nutrient concentrations in water [19]. Additionally, salinity increases, driven by climate change, have profound effects on eukaryotic microbial diversity and food web dynamics, leading to changes in the complexity and stability of ecological networks [26]. While extensive research has been conducted on the impacts of individual factors, such as temperature, precipitation, and salinity, on microbial diversity and composition in lake ecosystems, integrated studies addressing the combined effects of a warm‐wet climate remain scarce. This knowledge gap limits our understanding of how microbial diversity, community and network structural stability, and functional characteristics collectively respond to climate change. It also hinders efforts to elucidate the relationships among diversity, stability, and function, which are essential for predicting ecosystem resilience and adaptive responses under changing environmental conditions.

To bridge the above knowledge gaps, we conducted a comprehensive study involving long‐term field observations and mesocosm experiments with the following objectives (Figure 1): (1) Meteorological and aquatic environmental monitoring: We analyzed long‐term meteorological data from the Lake Bosten watershed (1958−2022) and integrated recent aquatic environmental parameters from Lake Bosten during the warm‐dry (2010−2011) and warm‐wet (2021−2022) periods, building on previous studies [9]. (2) Microbial community analysis: Using amplicon and metagenomic sequencing, we characterized bacterial and eukaryotic microbial communities in water samples collected during two distinct periods, separated by 10 years, from 17 monitoring sites across Lake Bosten (Figure S1 and Table S1). (3) Mesocosm simulation experiment: To better understand how microbial communities might respond to future climate change scenarios, we conducted a controlled mesocosm experiment over a 6 week period, crossing three temperature levels with three coupled hydrochemical scenarios involving paired salinity and nutrient conditions. Overall, this study aims to answer the following core scientific questions: (1) How does a warm‐wet hydroclimatic transition alter the aquatic environment of Lake Bosten, and which environmental factors drive microbial responses? (2) How do bacterial and eukaryotic communities respond in terms of diversity, composition, assembly processes, community compositional stability, and network structural stability? (3) How does this hydroclimatic transition reshape microbial functional potential, and what mechanisms underlie functional buffering under warm‐wet conditions?

**Figure 1:**
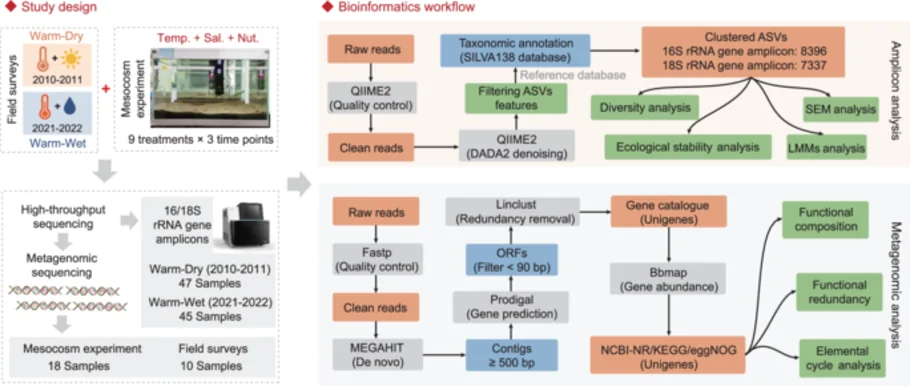
Study design and bioinformatics workflow. The study design consisted of two integrated components. First, field surveys were conducted during two contrasting hydroclimatic periods, namely the warm‐dry period (2010−2011) and the warm‐wet period (2021−2022), with 47 and 45 water samples collected, respectively, for 16S and 18S rRNA gene amplicon sequencing. Second, a mesocosm experiment comprising nine treatments and three sampling times was conducted to examine microbial responses to projected future environmental scenarios. A subset of samples, including 10 field samples and 18 mesocosm samples, was selected for metagenomic sequencing. The bioinformatics workflow includes two main components: amplicon analysis and metagenomic analysis, which were used to characterize microbial taxonomic composition, diversity, community compositional stability, network structural stability, functional composition, functional redundancy, and elemental cycling potential. ASVs, amplicon sequence variants; LMMs, linear mixed models; Nut., nutrients; ORFs, open reading frames; Sal., salinity; SEM, structural equation modeling; Temp., temperature.

## 2 RESULTS

### General characteristics and environmental dynamics of Lake Bosten

Long‐term meteorological records indicate pronounced climatic variability in the Lake Bosten basin over the past six decades (Figure 2A,B). Since 1958, air temperature at the Yanqi (YQ) station has shown a fluctuating but overall increasing trend, whereas temperatures at the Bayanbulak (BYB) station exhibited a clear upward shift after the late 1980s (Figure 2B). Precipitation at both stations declined between 1958 and 1986; however, precipitation at BYB increased again after 1987, consistent with the regional transition from a warm‐dry to a warm‐wet climatic regime.

**Figure 2:**
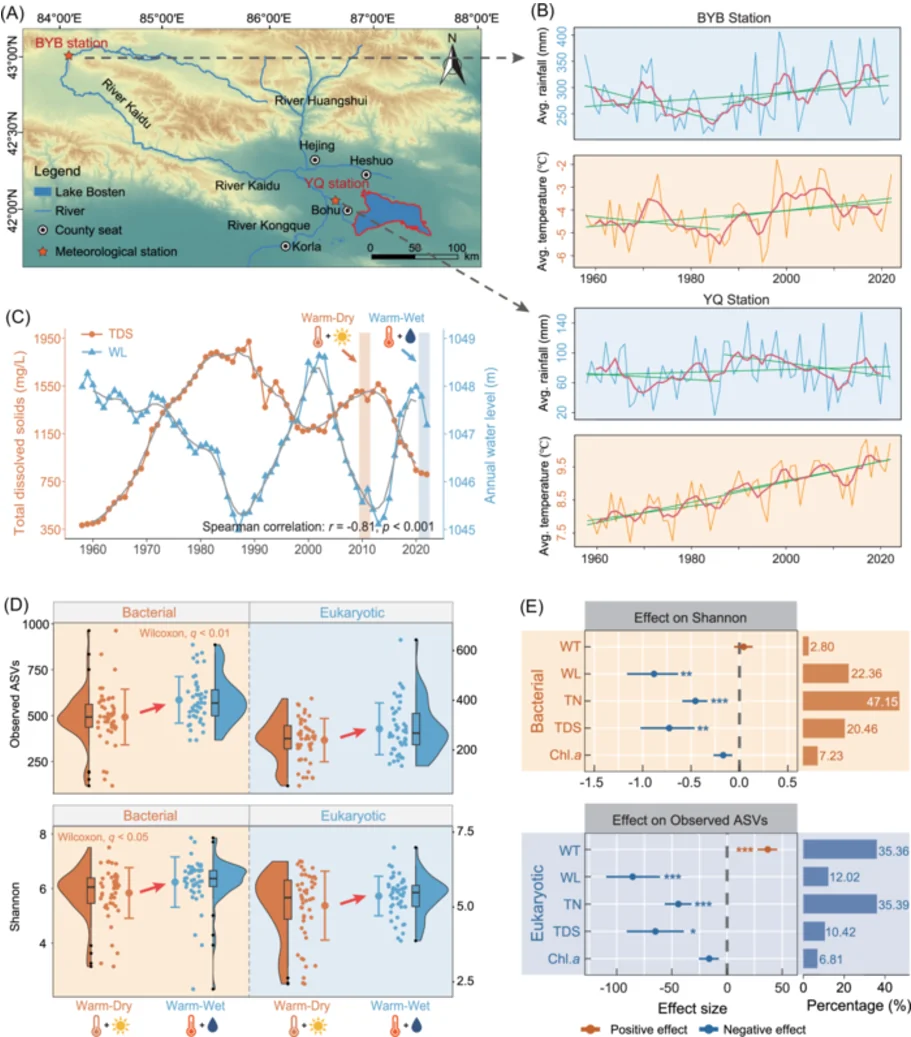
Long‐term meteorological, aquatic environmental dynamics, and microbial diversity in Lake Bosten. (A) Map of the Lake Bosten Basin. (B) Historical time series of annual mean air temperature and precipitation (orange and blue curves, respectively), 5 year moving averages (red curves), and their linear trends (green lines) for the Bayanbulak (BYB) and Yanqi (YQ) meteorological stations, covering the periods 1958−1986 and 1987−2022, respectively. Data source: China Meteorological Data Service Center (http://data.cma.cn/). (C) Trends in annual total dissolved solids (TDS; circles) and water level (WL; triangles) in Lake Bosten over 65 years. The relationship between TDS and WL was assessed using Spearman's rank correlation, followed by Benjamini–Hochberg FDR correction, with *q* < 0.05 considered significant. (D) Bacterial and eukaryotic microbial diversity during warm‐dry (WD) and warm‐wet (WW) periods. Each point represents microbial diversity metrics from a single water sample collected at a specific lake site and date, either during the warm‐dry period (2010−2011, *n* = 47) or the warm‐wet period (2021−2022, *n* = 45). Sampling locations and temporal coverage are detailed in Figure S1 and Table S1. The tops of the boxes represent the 75th percentile, the bottoms indicate the 25th percentile, and the center lines denote the median. The whiskers extend to the maximum and minimum non‐outlier values. (E) Effects of key environmental variables on microbial diversity, represented by regression coefficients (mean ± s.e.m.) from linear mixed effects models (*n* = 92). Positive coefficients indicate a positive association, whereas negative coefficients reflect suppressive effects on diversity. Significance: ***p < 0.001, **p < 0.01, *p < 0.05. Chl.*a*, chlorophyll‐*a*; TDS, total dissolved solids; TN, total nitrogen; WL, water level; WT, water temperature.

These climatic shifts were accompanied by pronounced hydrological and hydrochemical variability in Lake Bosten, reflected in long‐term fluctuations of water level (WL) and TDS (Figure 2C). WL exhibited distinct multi‐decadal oscillations. From 1958 to 1987, WL declined continuously from 1048.0 to 1045.0 m, with a corresponding reduction in water volume from 84.1 × 10^8^ to 53.7 × 10^8^ m^3^. This trend reversed between 1987 and 2002, with WL rising to 23 cm/year, peaking at 1048.7 m. A rapid decline followed from 2003 to 2013, reaching a minimum of 1045.1 m, before recovering steadily to 1047.9 m in 2019 and declining slightly thereafter.

In contrast to WL, TDS concentrations (i.e., salinity) demonstrated an inverse trend over the same period (Figure 2C). From 1958 to 1989, TDS levels increased dramatically from 0.39 to 1.93 g/L. During 1990−2003, TDS displayed a fluctuating decline, yet remained above 1 g/L. From 2004 to 2013, TDS concentrations rose again, reaching 1.57 g/L in 2013. However, in recent years, a marked decrease in TDS has been observed, with the lowest recorded value of 0.82 g/L in 2022. The co‐variation of WL and TDS thus represents a defining hydro‐chemical signal of environmental change in Lake Bosten.

Based on these hydrological, hydrochemical dynamics, and the climatic context, two contrasting environmental states were selected for microbial analyses: a WD period and a WW period. WL was strongly correlated with key water chemistry variables, especially TDS and total nitrogen (TN) (Figure S2). Additional environmental parameters also differed between the two climatic phases: during the WW period, water temperature (WT) and chlorophyll‐*a* (Chl.*a*) were significantly higher, whereas TDS, electrical conductivity (EC), and TN were lower relative to the WD period (Figure S3).

### Microbial diversity patterns from warm‐dry to warm‐wet climatic regimes

Bacterial Observed ASVs and Shannon diversity were significantly higher during the WW period than during the WD period after Benjamini–Hochberg FDR correction (Observed ASVs: *q* = 0.006; Shannon: *q* = 0.015), whereas Faith's PD showed a higher mean value but was not significant after correction (*q* = 0.117; Figure 2D and S4). Linear mixed effects models (LMMs) revealed that TN, WL, and TDS had significant negative effects on the Shannon diversity, explaining 47.2%, 22.4%, and 20.5% of the observed variation, respectively (Figure 2E). For eukaryotic microbes, diversity indices did not show significant differences between the WW and WD periods, but their average values were higher during the WW period (Figure 2D and Figure S4). According to LMMs, TN (*p* < 0.001), TDS (*p* < 0.05), and WL (*p* < 0.001) were identified as significant negative predictors of diversity (Figure 2E and Figure S5), with both TN and WT contributing about 35.4% to the explained variation in Observed ASVs (Figure 2E).

To investigate microbial diversity responses under projected climate‐driven environmental conditions, we conducted a controlled 40 day mesocosm experiment in which three temperature levels were crossed with three hydrochemical scenarios: control, dry associated, and wet associated conditions (Table S2). The nine treatment abbreviations were defined as follows: T_Ambient_ represented ambient treatments; T_Dry_ and T_Wet_ represented ambient temperature treatments with dry associated and wet associated hydrochemistry, respectively; T_Warm1_ and T_Warm2_ represented +3°C and +6°C warming with control hydrochemistry, respectively; T_Warm‐dry1_ and T_Warm‐wet1_ represented +3°C warming combined with dry associated and wet associated hydrochemistry, respectively; and T_Warm‐dry2_ and T_Warm‐wet2_ represented +6°C warming combined with dry associated and wet associated hydrochemistry, respectively. Because salinity and nutrient levels were intentionally paired within the dry and wet scenarios to represent ecologically plausible future hydrochemical conditions, the mesocosm design did not allow their independent effects to be separated. We therefore used range analysis to compare the relative response magnitude of microbial diversity to temperature versus the coupled hydrochemical scenario. Notably, the nutrient settings in the simulated future scenarios were based on projected long‐term conditions and therefore differed from the short‐term field contrast between the two sampling periods, in which the warm‐wet period exhibited lower nutrient concentrations than the warm‐dry period.

For bacteria, the range across temperature levels was larger than that across hydrochemical scenarios for both Observed ASVs and Shannon diversity (109.7 vs. 70.3 and 0.5 vs. 0.3, respectively; Table S3), suggesting that bacterial diversity was more responsive to warming within this experimental design. Under non‐warming conditions (T_Ambient_, T_Dry_, and T_Wet_), bacterial diversity tended to be higher under the wet hydrochemical scenario than under the control and dry scenarios (Figure S6A−C). Conversely, under future warm‐dry scenarios (T_Warm‐dry1_ and T_Warm‐dry2_), the combined effects of elevated temperature and dry hydrochemistry led to lower bacterial Observed ASVs than under non‐warming conditions. Under the +3°C warming level, the future warm‐wet scenario (T_Warm‐wet1_) partially increased bacterial diversity relative to the corresponding warm‐dry scenario (T_Warm‐dry2_) (Figure S6A−C).

For eukaryotic microbes, the range across hydrochemical scenarios exceeded that across temperature levels for both Observed ASVs and Shannon diversity (51.5 vs. 26.8 and 0.6 vs. 0.3, respectively; Table S4), indicating greater sensitivity to the coupled salinity‐nutrient shift within this experimental design. Comparisons between T_Warm‐dry1_ and T_Warm‐wet1_, as well as T_Warm‐dry2_ and T_Warm‐wet2_, suggested that wet hydrochemistry was associated with moderately higher eukaryotic diversity under warming conditions (Figure S6D−F). Nevertheless, eukaryotic diversity remained consistently lower in warming scenarios compared to ambient temperature conditions (T_Ambient_), indicating that wet hydrochemistry could only partially offset the adverse effects of warming.

‌Overall, the mesocosm experiment provided complementary evidence that bacterial and eukaryotic microbial diversity responded differently to warming and coupled dry/wet hydrochemical changes. Field observations identified WL, TDS, and TN as dominant bacterial diversity drivers, whereas the mesocosm experiment indicated that bacterial diversity was more responsive to temperature variation across the simulated future scenarios. For eukaryotes, field data identified WL, WT, and TN as key drivers, while the mesocosm experiment showed a stronger response to coupled hydrochemical scenarios than to temperature. Because salinity and nutrients were not independently manipulated, these mesocosm results should be interpreted as scenario based responses rather than as separate causal effects of salinity and nutrients.

### Microbial community composition from warm‐dry to warm‐wet climatic regimes

Principal coordinate analysis (PCoA) revealed significant differences in microbial communities between the WD and WW periods (PERMANOVA: *p* < 0.001; Figure S7). For bacteria, community structure exhibited higher similarity among sites during the WW period compared to the WD period. At the phylum level (Figure 3A,B), heterotrophic taxa dominated the bacterial community, particularly Proteobacteria, Actinobacteriota, and Verrucomicrobiota. The relative abundance of Verrucomicrobiota increased from 14.6% to 30.2% during the transition from WD to WW, whereas Proteobacteria (31.2% to 26.3%) and Firmicutes (9.5% to 1.8%) showed opposite trends. The photoautotrophic Cyanobacteria remained relatively stable across the two climatic periods (9.0% to 7.6%). At the genus level (Figure S8), *Luteolibacter* (3.0% to 11.5%) and the hgcI clade (3.6% to 6.8%) exhibited increased relative abundances from WD to WW, while *Brevundimonas* (8.1% to 3.4%), *Exiguobacterium* (5.4% to 1.4%), *Microbacterium* (2.6% to 0.08%), and *Candidatus Aquiluna* (3.0% to 1.6%) decreased. These shifts in bacterial community composition were primarily driven by TN (36.1%), TDS (21.4%), and WL (18.9%) (Figure 3C and Figure S9). Regarding ecological assembly processes, stochastic mechanisms, particularly drift and dispersal limitation, were the dominant drivers of bacterial community structure. However, from the WD to the WW period, the contribution of homogeneous selection increased from 33.1% to 37.2%. Concurrently, the influence of dispersal limitation within stochastic processes declined from 41.5% to 26.1% (Figure 3D).

**Figure 3:**
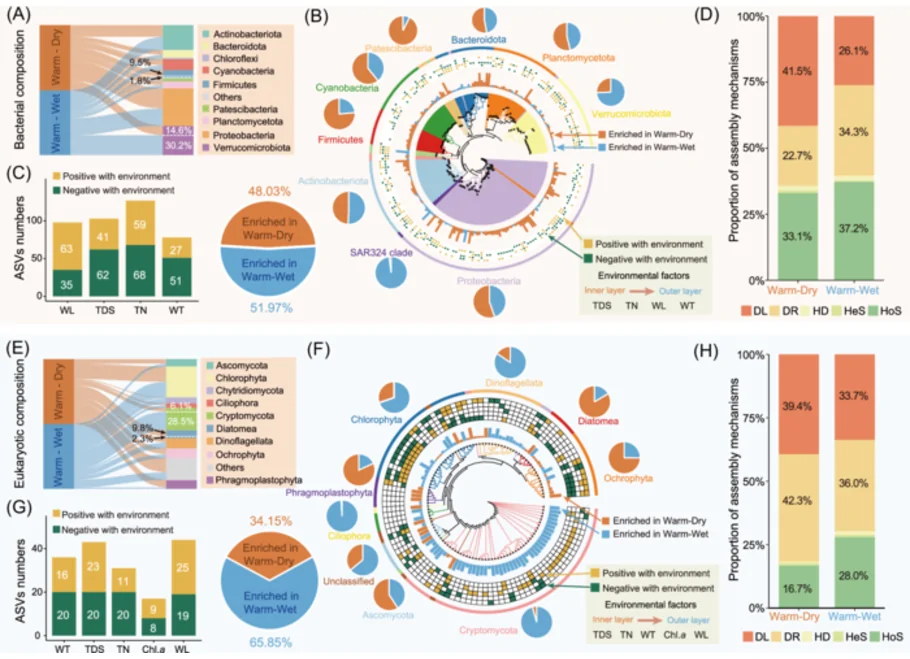
Mechanisms underlying shifts in bacterial and eukaryotic communities during warm‐dry (WD) and warm‐wet (WW) periods. (A) Bacterial community composition during the WD and WW periods. Only the top 9 phyla are shown, and all remaining phyla are grouped as “others.” (B) Phylogenetic tree of bacterial ASVs, including only ASVs that showed significant responses to climate conditions based on DESeq2 analysis after Benjamini–Hochberg FDR correction (*q* < 0.05) and had mean relative abundances > 0.05%. The first and fourth rings represent phylum level taxonomy. The second ring indicates the direction of ASV change under climatic shifts, and the third ring denotes significant correlations between ASVs and physicochemical variables. (C) Numbers and proportions of bacterial ASVs significantly associated with specific environmental parameters and enriched during the WD or WW period. (D) Community assembly mechanisms of bacterial communities based on a phylogenetic bin based null model implemented using the “iCAMP” R package. (E) Eukaryotic community composition during the WD and WW periods. Only the top 9 phyla are shown, and all remaining phyla are grouped as “others.” (F) Phylogenetic tree of eukaryotic ASVs, including only ASVs that showed significant responses to climate conditions based on DESeq2 analysis after Benjamini–Hochberg FDR correction (*q* < 0.05) and had mean relative abundances > 0.2%. The first and fourth rings represent phylum level taxonomy. The second ring indicates the direction of ASV change under climatic shifts, and the third ring denotes significant correlations between ASVs and physicochemical variables. (G) Numbers and proportions of eukaryotic ASVs significantly associated with specific environmental parameters and enriched during the WD or WW period. (H) Community assembly mechanisms of eukaryotic communities based on a phylogenetic bin based null model implemented using the “iCAMP” R package. ASV, amplicon sequence variant; HeS, heterogeneous selection; HoS, homogeneous selection; DL, dispersal limitation; DR, drift; HD, homogenizing dispersal; iCAMP, infer community assembly mechanisms by phylogenetic bin based null model analysis.

For eukaryotic microbial communities, phylum level patterns (Figure 3E,F) showed that photoautotrophic Chlorophyta, Dinoflagellata, and Diatomea, together with heterotrophic Cryptomycota, were the dominant groups. The relative abundance of Cryptomycota increased sharply from 6.1% to 28.5% during the transition from WD to WW, whereas Diatomea (9.8% to 2.3%) showed opposite trends. Although photoautotrophic taxa maintained relatively high overall abundance, Chlorophyta (23.6% to 24.0%), Dinoflagellata (7.7% to 8.9%), and Ochrophyta (9.3% to 6.1%) remained relatively stable across climate periods. At the genus level (Figure S18), *LKM11* exhibited a substantial increase in relative abundance (4.3% to 28.3%) during the WW period, while unidentified Chlorophyceae (17.6% to 10.8%) and *Aspergillus* (3.6% to 0.2%) declined. Approximately 65.9% of dominant phylotypes showed an increase in abundance during WW (Figure 3F), with changes significantly correlated with TDS, WL, WT, TN, and Chl.*a* (Figure 3G). Similar to bacterial community, stochastic processes, primarily dispersal limitation and drift, were the dominant forces shaping eukaryotic microbial community assembly. However, from the WD to the WW period, the contribution of homogeneous selection increased from 16.7% to 28.0%. In parallel, the influence of dispersal limitation within stochastic processes declined from 39.4% to 33.7% (Figure 3H).

As a result, the shift from WD to WW induced significant microbial community restructuring, with bacterial and eukaryotic taxa displaying distinct but overlapping environmental drivers and assembly mechanisms.

### Community and network stability from warm‐dry to warm‐wet climatic regimes

Bacterial and eukaryotic microbes exhibited distinct network patterns (Figure 4A,B) and topological properties (Table S5) during the WD and WW periods. For bacterial ecological networks, network complexity, evaluated by complexity index, average degree (AvgK), and connectedness, significantly decreased from WD to WW. Community compositional stability, assessed by average variation degree (AVD), also declined, as indicated by higher AVD values during the WW period. In parallel, network structural stability, assessed by robustness and vulnerability, decreased from WD to WW, with lower robustness and higher vulnerability. Moreover, the number of keystone taxa decreased from 7 in the WD to 5 in the WW period (Figure S10 and Table S6). Similar patterns were observed in eukaryotic microbial networks. Collectively, both bacterial and eukaryotic communities showed reduced compositional stability and lower network structural stability from the WD to the WW period.

**Figure 4:**
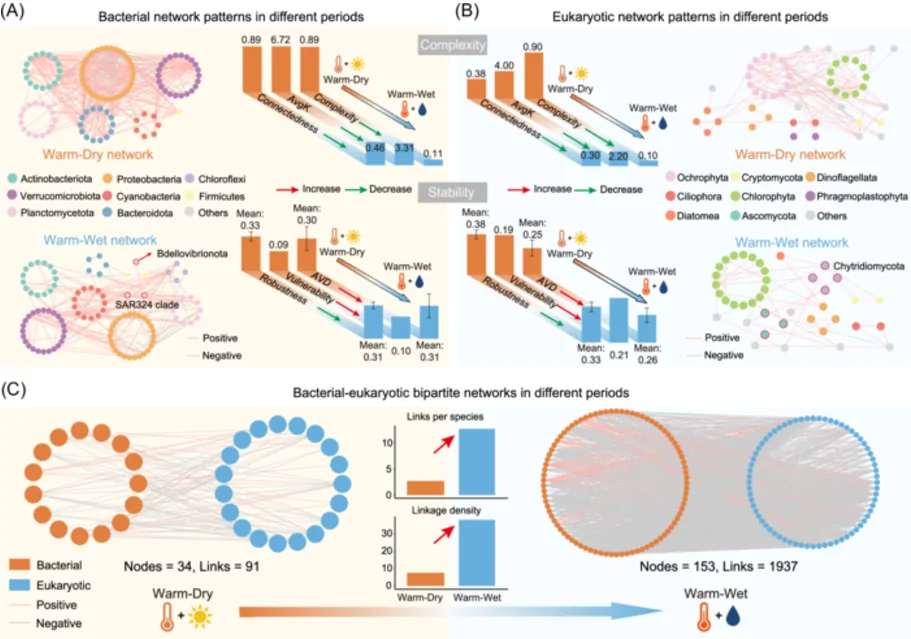
Bacterial, eukaryotic, and bacterial‐eukaryotic bipartite networks during warm‐dry (WD) and warm‐wet (WW) periods. (A) Bacterial co‐occurrence network patterns and associated network complexity and stability metrics. (B) Eukaryotic microbial co‐occurrence network patterns and associated network complexity and stability metrics. (C) Bacterial‐eukaryotic bipartite networks, showing changes in network size, link number, links per species, and linkage density. Microbial co‐occurrence networks were constructed using Spearman's correlation coefficients calculated via the Integrated Network Analysis Pipeline (iNAP, https://inap.denglab.org.cn/). Network construction employed similarity thresholds determined by random matrix theory (RMT): 0.81 for bacterial networks and 0.70 for eukaryotic networks. Network complexity was assessed based on three key metrics: (1) a composite complexity index, (2) average degree (avgK), and (3) connectedness. The complexity index was calculated as the mean of standardized values for multiple network parameters, including the number of nodes, the number of links, avgK, average clustering coefficient (AvgCC), average path distance (GD), geodesic efficiency (GE), centralization of betweenness, centralization of stress centrality, density, and connectedness. Community compositional stability was measured using average variation degree (AVD), whereas network structural stability was evaluated using robustness and vulnerability.

In contrast, bipartite networks linking bacterial and eukaryotic microbes revealed divergent patterns (Figure 4C). Under the WD period, the bipartite network comprised 34 nodes and 91 links, whereas under the WW period, it expanded to 153 nodes and 1937 links. Both links per species and linkage density significantly increased from WD to WW, and the number of keystone taxa in the bipartite network rose markedly from 11 in WD to 59 in WW (Figure S11 and Table S7).

### Microbial functional adaptations from warm‐dry to warm‐wet climatic regimes

Microbial functional composition was represented by KEGG pathways and COG categories from the eggNOG database. PCoA analysis showed that functional composition was more similar among samples during the WW period than during the WD period, but no significant period shift in broad functional composition was detected (all PERMANOVA: *p* > 0.05; Figure S12). Consistent with this pattern, the relative abundance distributions of several major functional categories, including methane metabolism, carbon fixation, central metabolism, oxidative phosphorylation, and sulfur metabolism, showed only minor differences between the two periods (Figure S13). Because the field metagenomic dataset included a limited number of samples, these results were interpreted as an absence of detected broad functional shifts rather than as definitive evidence of functional stability. Redundancy analysis (RDA) revealed that functional composition during both the WD and WW periods was primarily associated with TN (KEGG: 45.4%, COG: 25.2%), TDS (KEGG: 18.2%, COG: 8.9%), Chl.*a* (KEGG: 18.9%), WT (COG: 27.7%), and COD (COG: 36.3%) (Figure S9).

Although the overall functional composition did not differ significantly between the WD and WW periods, the number of enriched functional genes, defined as genes showing significantly higher relative abundance in one period compared to the other, was notably higher during the WW period. Based on KEGG functional annotations, 146 genes were enriched during the WD period, including 33 highly enriched and 113 moderately enriched genes. In contrast, 221 genes exhibited higher relative abundance during the WW period, comprising 71 highly enriched and 150 moderately enriched genes. Similarly, COG functional annotations revealed 341 genes enriched under WD conditions (142 highly enriched and 199 moderately enriched), while 371 genes were enriched under WW conditions (139 highly enriched and 232 moderately enriched) (Figure 5A).

**Figure 5:**
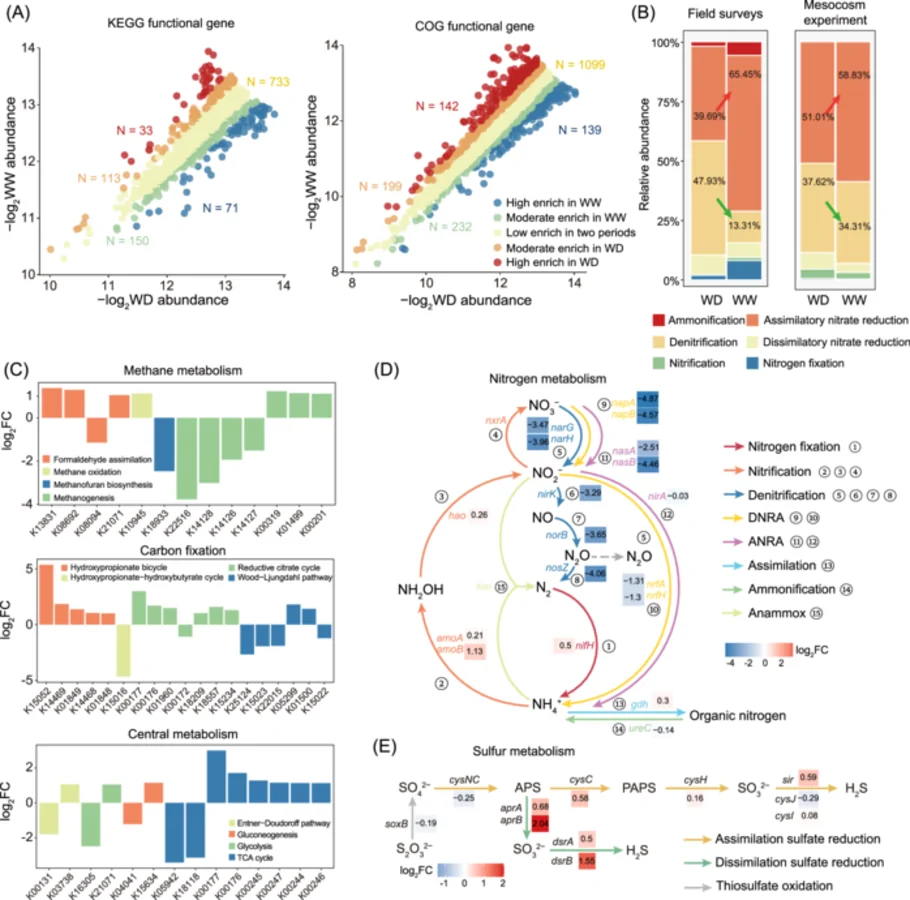
Microbial functional adaptations during warm‐dry (WD) and warm‐wet (WW) periods. (A) Volcano plots showing relative abundance changes in KEGG (1100 genes) and COG (1811 genes) annotations across the two climate periods in field surveys. Only genes with relative abundance > 0.01% were included. Relative abundances were −log_2_‐transformed fold change (−log_2_FC) between the WW and WD periods. Genes were classified as highly enriched (|log_2_FC| > 0.5), moderately enriched (0.25 < |log_2_FC| ≤ 0.5), or lowly enriched (|log_2_FC| ≤ 0.25). (B) Functional composition of nitrogen metabolism in both field surveys and mesocosm experiments. (C) Functional differences in methane metabolism, carbon fixation, and central metabolism across climate periods in field surveys. (D, E) Heatmaps showing log_2_ fold change (log_2_FC) in gene abundance related to nitrogen and sulfur cycling in field surveys. Positive log_2_FC values indicate gene enrichment during the WW period; negative values indicate higher abundance during the WD period. Values exceeding |1| represent at least a twofold change. ANRA, assimilatory nitrate reduction to ammonium; APS, adenosine 5'‐phosphosulfate; DNRA, dissimilatory nitrate reduction to ammonium; PAPS, 3'‐phosphoadenosine 5'‐phosphosulfate.

Nitrogen metabolism exhibited the most striking climate phase differences. The proportion of genes associated with assimilatory nitrate reduction increased markedly from 39.7% during the WD period to 65.5% during the WW period (Figure 5B), while the relative proportion of denitrification related genes declined sharply from 47.9% to 13.3% during the same period (Figure 5B). In the mesocosm experiment, the projected future WW scenario was defined by lower salinity but higher nutrient concentrations, which differed from the lower TN observed during the contemporary WW field period. Despite these distinct nutrient backgrounds, the WW treatments showed the same directional shift in nitrogen functional composition as the field survey, with a higher relative contribution of assimilatory nitrate reduction and lower denitrification (Figure 5B). This agreement indicates a convergent functional pattern under WW related conditions, but should not be interpreted as evidence that opposite TN changes exerted the same direct effect.

Other pathways also displayed phase‐specific changes. In methane metabolism, relative abundance involved in genes of formaldehyde assimilation (K13831, K08692, K21071) and methane oxidation (K10945) were higher during the WW period, whereas relative abundances of genes related to methanofuran biosynthesis (K18933) were higher in the WD period (Figure 5C). For carbon fixation, the relative abundances of genes associated with the 3‐hydroxypropionate bi‐cycle were increased in the WW period (Figure 5C). In terms of central metabolism, the number of genes related to the tricarboxylic acid (TCA) cycle pathway was more abundant during the WW period compared to the WD period (Figure 5C). Additional nitrogen metabolism genes showed climate phase specificity. Genes associated with nitrate reduction and denitrification, including *napA*, *napB*, *narG*, *narH*, *nirK*, *nirS*, *norB*, and *nosZ*, generally showed lower abundances during the WW period. In contrast, genes associated with nitrogen fixation and selected nitrification or ammonification processes, including *nifH*, *amoA*, *amoB*, *hao*, *norC*, and *gdh*, showed higher abundances during the WW period (Figure 5D and Table S8). The relative abundances of sulfur metabolism genes related to dissimilatory sulfate reduction (*aprB*, *dsrB*) were increased during the WW period (Figure 5E).

Collectively, although no significant period shift in broad functional composition was detected, specific nitrogen cycling genes showed pathway‐dependent redistribution. Genes related to nitrogen fixation and selected nitrification or ammonification processes were higher during the WW period, whereas genes associated with nitrate reduction, denitrification, and DNRA were generally lower. These results indicate that microbial nitrogen metabolic potential was reorganized rather than uniformly enhanced under WW conditions.

### Interrelationships among microbial diversity, community compositional stability, and functional traits

The structural equation modeling (SEM) was used as an exploratory framework to summarize hypothesized associations among climate conditions, aquatic environmental variables, microbial diversity, community compositional stability, functional redundancy, and predicted functional traits (Figure 6A). Climate conditions showed relatively weak direct associations with microbial diversity (*r* = −0.17) and functional redundancy (*r* = −0.12). Environmental conditions associated with the warm‐wet period, including higher WL and lower TN and TDS, were positively linked to microbial diversity. Microbial diversity was further associated with lower community compositional stability (Figure 6B), and higher functional redundancy (Figure 6C). Moreover, microbial diversity (*r* = −0.75, *p* < 0.001) and community compositional stability (*r* = −0.41, *p* < 0.001) were negatively correlated with functional traits (Figure 6A,D). These patterns suggest that microbial diversity, compositional stability, functional redundancy, and predicted functional traits were tightly coupled during the warm‐dry to warm‐wet transition.

**Figure 6:**
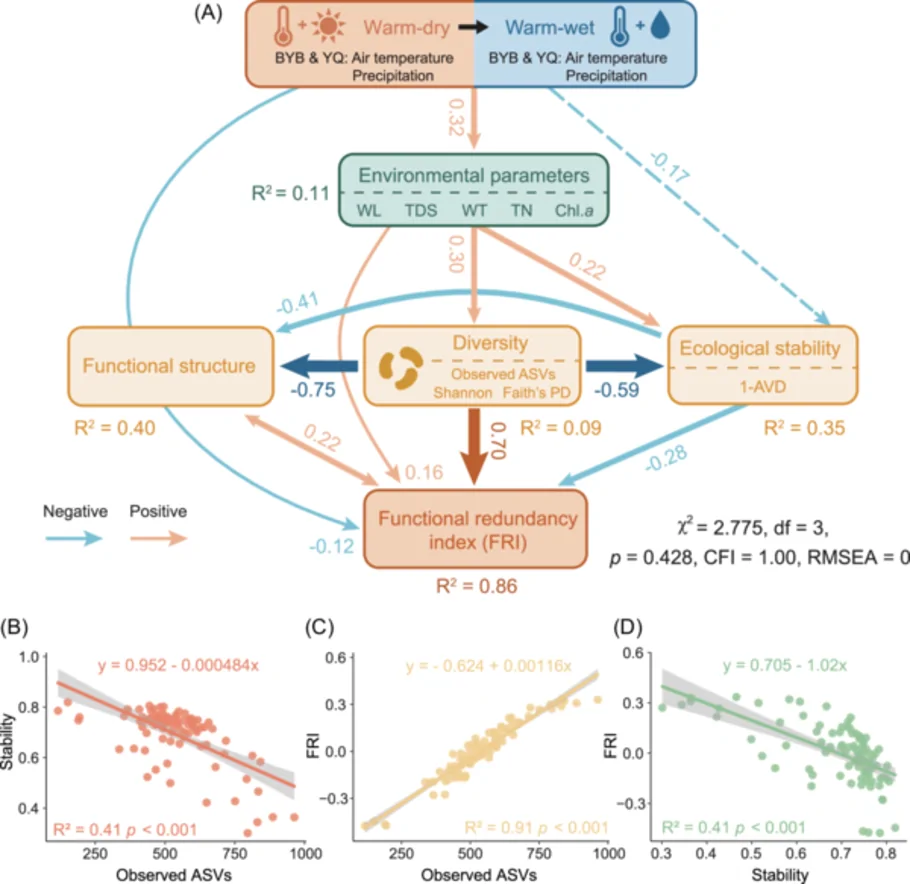
Microbial response patterns to warm‐wet climate. (A) Structural equation model (SEM) depicting the relationships among climate change, environmental parameters, diversity, community compositional stability, and function. Blue arrows indicate negative relationships, red arrows positive relationships; solid and dashed lines represent significant (*p* < 0.05) and non‐significant paths, respectively. Standardized path coefficients and R^2^ values (variance explained) are shown. (B−D) Univariate linear regressions illustrating the relationships among Observed ASVs, community compositional stability, and FRI. ASV, amplicon sequence variant; AVD, average variation degree; FRI, functional redundancy index; BYB, Bayanbulak; CFI, comparative fit index; Chl.*a*, chlorophyll‐*a*; df, degrees of freedom; RMSEA, root mean square error of approximation; TN, total nitrogen; TDS, total dissolved solids; WT, water temperature; WL, water level; *χ* ^2^, chi‐square statistic; YQ, Yanqi.

## 3 DISCUSSION

To our knowledge, this study provides the first comprehensive assessment of how a shift from a warm‐dry to warm‐wet climate reshapes the diversity, stability, and functions of microbial communities in a lake ecosystem of Northwest China. Overall, bacterial and eukaryotic microbial communities showed partly similar responses to the climatic shift. Both groups exhibited higher diversity and lower network complexity, accompanied by reduced community or network structural stability. The climatic shift also increased functional redundancy and promoted structural convergence across microbial groups. At the functional level, genes related to nitrogen fixation, and assimilatory nitrate reduction were enriched.

Mechanistically, WW conditions affected Lake Bosten through two main pathways. The first was direct warming. The second was WL change, which altered hydrological connectivity and indirectly reshaped water chemistry, including salinity and nutrients.

Global warming poses a serious threat to microbial diversity [22, 23, 27]. In our previous studies, simulated warming reduced both bacterial and eukaryotic diversity in Lake Bosten [25, 28], consistent with the current mesocosm results (T_Ambient_, T_Warm1_, and T_Warm2_; Figure S6, Tables S3−4). The observed loss of microbial diversity under warming may result from changes in both abiotic conditions and biotic interactions [25, 27, 28]. According to niche theory, elevated temperatures can act as a deterministic filter that favors thermotolerant taxa while reducing or eliminating cold adapted species, ultimately leading to a decline in overall diversity [25, 27]. In addition, warming alters species interactions across multiple trophic levels, potentially triggering local extinctions, reducing network complexity, and initiating cascading effects that further erode microbial diversity [25, 29].

Beyond temperature, WL emerged as a central explanatory factor for microbial diversity, and community assembly. First, WL serves as a quantitative proxy for hydrological connectivity [30, 31]. In Lake Bosten, WL is not simply a vertical measure of water depth; rather, it approximates ecosystem‐wide connectivity forced by climate variability. Higher WL reconnects previously isolated habitats, reduces dispersal barriers, and enhances horizontal microbial migration, thereby modulating habitat connectivity, dispersal opportunities, and resource availability [32, 33]. Although water depth is often used as a local physical descriptor, and the two parameters may coincide at a given location, water depth only reflects vertical conditions at that site [34, 35]. In contrast, WL integrates system‐level hydrological connectivity over climatic timescales (i.e., between the warm‐dry and warm‐wet climatic regimes) [36, 37]. This broader dimension of WL, representing lake‐wide dispersal and habitat reconnection, cannot be captured by water depth alone. Therefore, our findings highlight that WL functions as a system level driver shaping microbial communities through both physical connectivity and environmental homogenization.

Second, WL significantly shaped community structure and assembly. Field analyses identified WL (together with TDS and TN) as dominant drivers of bacterial diversity, and WL, WT, and TN as dominant drivers for eukaryotes (Figure 2E). Assembly analysis further revealed that, from WD to WW, the contribution of homogeneous selection increased, while dispersal limitation within stochastic processes declined (Figure 3D,H). These findings are consistent with WL‐driven increases in hydrological connectivity: as habitats become reconnected under higher WL, dispersal constraints weaken, environmental filters become more uniform at the basin scale, and microbial communities converge structurally.

Finally, WL adds a unique dimension beyond chemistry. Although WL correlates with chemical proxies such as TDS and TN (Figure S2), it is not redundant with them. WL integrates both (i) the physical connectivity that governs dispersal and selection across space and (ii) the chemical dilution that alleviates stress. We observed significantly reduced TDS (from oligo‐saline toward freshwater) and lower TN during WW (Figures S2−3), indicating that wetting induced WL further decreased TDS and TN, thereby enhancing microbial diversity and partially offsetting the negative impacts of warming.

Collectively, WL functions as an integrative, system level mechanism that (i) reflects climate induced hydrological shifts, (ii) modulates hydrological connectivity and dispersal processes, and (iii) indirectly reshapes microbial habitats via its effects on TDS and TN.

Salinity is widely recognized as a key environmental stressor for microbial communities [26, 38]. As salinity increases, elevated extracellular osmotic pressure [19, 38] and changes in reactive oxygen species dynamics [39] can constrain microbial niche breadth and ultimately reduce community diversity. Declines in microbial diversity with increasing salinity are commonly observed in lake ecosystems [26, 40]. For example, in the Plateau Lakes of the Tibetan Plateau, bacterial diversity decreases significantly with rising salinity, and the relative abundance of Verrucomicrobiota has been shown to be negatively correlated with salinity [40]. Similar patterns have been reported for microeukaryotic communities in lakes of the Inner Mongolia–Xinjiang Plateau [26]. In the present study, lower salinity during the WW period was associated with higher microbial diversity. Verrucomicrobiota increased from 14.6% to 30.2%, and Cryptomycota from 6.1% to 28.5%, between the WD and WW periods. Similarly, the proportion of enriched microeukaryotic ASVs increased from 34.2% to 65.8%, suggesting increased taxonomic complexity under lower salinity conditions during the WW period. Additionally, environmental heterogeneity was higher in the WW period than in the WD period. This may be related to enhanced glacier melt and rainfall under warm‐wet conditions, which can transport more solutes into lakes through physical erosion. Increased resource availability may further promote microbial diversity. For instance, species absent in the WD period, such as Bdellovibrionota, SAR324 clade, Chytridiomycota, and Ascomycota, were found in the WW microbial co‐occurrence networks (Figure 4).

Bacterial and eukaryotic communities showed both shared and domain‐specific responses to the warm‐dry to warm‐wet transition. Both groups underwent pronounced compositional reorganization and showed parallel shifts in assembly processes, characterized by weaker dispersal limitation and stronger homogeneous selection under the WW regime. This convergence suggests that bacteria and eukaryotic microbes were jointly filtered by the same hydroclimatic changes, particularly the associated increase in WL and decline in salinity. However, their taxonomic responses were not synchronous. Bacterial restructuring was characterized mainly by the enrichment of Verrucomicrobiota and the decline of Firmicutes, whereas eukaryotic restructuring was marked by the increase in Cryptomycota and the decrease in Diatomea. Their diversity responses also differed, with a significant increase in bacterial diversity but only a weaker increase in eukaryotic diversity. These differences likely reflect contrasting ecological strategies between the two domains. Bacteria, with shorter generation times and broad metabolic flexibility, may respond rapidly to altered physicochemical conditions, whereas eukaryotic microbes are additionally shaped by trophic mode, food‐web interactions, life‐history traits, and habitat dependence [41, 42].

The functional responses were also not fully symmetrical between the two groups. Although both domains showed marked compositional turnover, the measured nitrogen cycling potential was mainly contributed by bacterial taxa in the field metagenomes. The major bacterial carriers of nitrogen cycling functions also shifted between the WD and WW periods (Figure S14). This indicates that warm‐wet hydroclimatic change generated a shared environmental filter across microbial domains, but the directly measured biogeochemical functional reorganization was expressed mainly through turnover within bacterial metabolic guilds. Thus, bacterial and eukaryotic communities were similar in their broad sensitivity to hydroclimatic forcing, yet differed in the taxa that responded and in their apparent linkage to the measured elemental cycling functions.

Microorganisms do not exist in isolation but interact to form complex microbial communities [43, 44, 45]. These interactions influence community composition, structural organization, and the distribution of ecological functions [45, 46, 47]. However, stability is a multidimensional concept. Classical biodiversity‐stability theory has often focused on the stability of aggregate ecosystem properties or functions [48, 49], whereas the stability metrics used in our study quantify community compositional variation and network structural robustness. In Lake Bosten, warm‐wet conditions increased microbial diversity but reduced community compositional stability and network structural stability. In contrast, no significant period shift in broad metagenomic functional composition was detected, suggesting that functional potential may have been comparatively buffered. Therefore, our findings should not be interpreted as a direct challenge to the classical diversity‐stability framework. Instead, they reveal a decoupling among taxonomic diversity, structural stability, and functional potential under hydroclimatic change.

First, under warm‐wet conditions, bacterial community composition and functional potential became more homogeneous, with a higher proportion of positive correlations in the ecological network, reducing niche differentiation and interspecies interaction diversity [50, 51]. This homogenization not only simplified network structure, reducing complexity, but also weakened community resilience to disturbances, ultimately reducing network structural stability [52].

Second, deterministic processes became more important under the warm‐wet climate. In particular, inferring community assembly mechanisms by phylogenetic bin based null model analysis (iCAMP) showed that homogeneous selection explained a larger proportion of community assembly during the WW period (Figure 3D,H). This shift reflects the influence of more homogeneous environmental pressures during the WW period, which likely contributed to greater taxonomic similarity across microbial communities [53]. In parallel, environmental filtering was also intensified. This was indicated by an increase in bacterial taxa significantly correlated with environmental parameters, from 195 to 212. These environmental filters favored specialized taxa well adapted to the prevailing conditions [19]. In the eukaryotic microbial network, this shift was reflected in the increased representation of Chlorophyta associated nodes and the disappearance of Ochrophyta nodes, further underscoring the influence of selective pressures. The resulting decline in interaction diversity led to a more simplified network, characterized by fewer nodes and links, and reduced overall complexity [50, 52].

Additionally, increased microbial diversity may promote a higher prevalence of weak or transient interactions in highly diverse communities, where some species interact only occasionally or weakly [46, 54]. For example, in the bacterial network, Bdellovibrionota had only one link, and the SAR324 clade had two links. In the eukaryotic microbial network, Ascomycota and Chytridiomycota appeared during the WW period but had few links with other species. These weakly connected taxa may dilute strong interdependencies, thereby reducing network complexity and weakening network robustness to environmental disturbances [46, 54].

Finally, theoretical studies predict that ecological networks composed of weak interactions are more stable than those dominated by strong interactions, and increased negative interactions enhance network stability under disturbances [51, 54]. In the eukaryotic microbial network, positive links increased from 93.8% in WD to 97.7% in WW. This pattern suggests higher niche overlap and/or stronger positive interspecies associations. While positive interactions can promote coexistence, they may also create positive feedback loops that destabilize microbial communities [50, 51]. When such interdependent relationships are disrupted, for instance, due to the loss of key species (Figure S10 and Table S6), cascading effects could severely impair community stability [52]. Meanwhile, we also observed accelerated community turnover in eukaryotic microbes, further contributing to reduced stability (Figure S15). Overall, warm‐wet conditions increased microbial diversity but simplified network architecture and weakened structural stability. At the same time, broad functional potential was maintained through functional redundancy and compensation.

The decline in within domain network structural stability should be interpreted together with the strengthened bacterial‐eukaryotic bipartite network. These two patterns reflect different levels of microbial organization rather than contradictory responses. Reduced robustness and increased vulnerability indicate weakened structural stability within bacterial or eukaryotic networks, whereas the strengthened bipartite network suggests enhanced cross domain coupling under WW conditions. Functional annotation of keystone taxa in the bacterial‐eukaryotic bipartite network further supported this interpretation (Table S7). In the bipartite network analysis, connector hub keystone taxa increased from 11 during the WD period to 59 during the WW period, indicating stronger cross domain connectivity under WW conditions. These keystone taxa were associated with complementary ecological roles, including bacterial organic matter degradation, C1 carbon transformation, nutrient regeneration, particle associated carbon turnover, and eukaryotic phototrophic production, grazing, parasitism, and food‐web regulation. Therefore, strengthened bacterial‐eukaryotic associations may form a compensatory interaction layer linking bacterial nutrient transformation and organic matter processing with eukaryotic production and trophic regulation. Such cross domain rewiring may contribute to functional buffering despite reduced within domain network structural stability. However, because co‐occurrence networks infer statistical associations rather than direct interactions, this compensatory mechanism should be interpreted as a plausible ecological linkage that requires further experimental validation.

Generally, climate warming and increased hydrological inputs are expected to alter the community composition of phytoplankton or photoautotrophs [55]. However, in Lake Bosten, the taxonomic compositions of photoautotrophic groups (Cyanobacteria, Dinoflagellata, Ochrophyta) remained relatively stable from the WD to the WW periods (Figures S16–17). This stability likely reflects two factors: (i) minimal differences in light intensity between the two periods (Figure S18) and (ii) persistent nutrient limitation, as the lake is mesotrophic with low long‐term total phosphorus (TP; ~ 0.003 mg/L) and further TN decline during the WW period. Under such conditions, warming may increase photoautotroph abundance (e.g., Chl.*a*; Figure S3) without changing community composition. Within this context, we observed only a slight increase in the relative abundance of Dinoflagellata, but their overall contribution to the community remained low, in contrast to the predominance of heterotrophic groups such as Cryptomycota. This pattern strongly suggests that bottom‐up nutrient limitation, rather than temperature alone, constrained compositional shifts in photoautotrophs [56, 57]. Consequently, nutrient limitation may explain the modest compositional changes in phototrophic groups. In contrast, larger climate linked shifts were concentrated in heterotrophic taxa, such as Verrucomicrobiota and Cryptomycota. These taxa contributed more strongly to changes in network architecture and community assembly.

Importantly, no significant period shift in broad metagenomic functional composition was detected between the WD and WW periods based on KEGG and COG annotations. This pattern was observed for several broad biogeochemical categories, including photosynthesis, carbon fixation, and oxidative phosphorylation. This apparent buffering of functional potential may reflect a high degree of microbial functional redundancy (Figure S19), whereby different taxa perform overlapping metabolic roles [58, 59, 60, 61, 62]. For example, compositional changes involving Verrucomicrobiota, Cryptomycota, Firmicutes, or Diatomea did not correspond with losses in core metabolic functions, suggesting that functionally equivalent taxa compensated for taxonomic turnover [61, 62]. The proposed functional compensation was pathway‐specific and was mainly mediated by taxonomic replacement within bacterial functional guilds. The nitrogen cycle pathway analysis showed that functional responses were not uniform across the nitrogen cycle. Nitrogen fixation and some nitrification genes increased under WW conditions, whereas nitrate reduction, nitrous oxide reduction, and DNRA genes generally showed lower abundance (Figure 5D and Table S8).

Taxon‐function linkage analysis further showed that the dominant contributors to the measured nitrogen cycling potential were mainly bacterial taxa, but the major carriers of these functions differed markedly between the WD and WW periods (Figure S14). For example, nitrogen fixation shifted from *Cereibacter*, *Stutzerimonas*, and *Alcaligenes* under WD conditions to unidentified genera within Burkholderiales, *Limnohabitans*, and *Vulcanococcus* under WW conditions. Nitric oxide reduction shifted from *Ochrobactrum*, *Brevundimonas*, and *Shinella* to *Rubritalea*, unidentified genera within Verrucomicrobiota, and Planctomycetota. In contrast, nitrite ammonification showed partial persistence of functional carriers, with unidentified genera within Planctomycetota remaining a major contributor, while *Hyphomonas* and other bacterial taxa increased under WW conditions. These patterns suggest that functional compensation was not species invariant or pathway universal, but instead reflected pathway‐specific redistribution among bacterial taxa carrying related nitrogen cycling genes. This finding is consistent with the concept of an “insurance effect”, whereby functionally equivalent taxa can buffer functional potential against taxonomic turnover under environmental fluctuations [59, 60, 62].

Lake Bosten is a mesotrophic, phosphorus limited system. The field survey and mesocosm experiment captured different temporal contexts of WW conditions. The contemporary field period was characterized by WL associated dilution and lower TN. In contrast, the projected future WW mesocosm scenario combined lower salinity with higher nutrient levels, representing the possibility that enhanced external loading may offset dilution under continued warming and wetting. Therefore, the two datasets should be viewed as complementary rather than directly matched tests of nutrient effects. Their convergent nitrogen functional pattern, with a higher relative contribution of assimilatory nitrate reduction and lower denitrification under WW related conditions, suggests that this functional reorganization may occur across different hydrochemical backgrounds. However, because the mesocosm experiment was scenario based and salinity and nutrients were coupled by design, the independent contribution of nutrient changes cannot be disentangled here.

This study focused on Lake Bosten, the largest lake in arid northwestern China. This focus was necessary because long‐term field observation data are limited for arid‐region lakes. However, the regional generalizability of our findings requires further validation by expanding the scope to include diverse lake types (e.g., alpine glacial lakes and closed saline lakes) and broader geographic regions (e.g., the Tibetan Plateau and the Central Asian arid zone). Nevertheless, our study provides insights for arid‐region lake management and climate adaptation. First, warm‐wet conditions enhanced microbial diversity by regulating the water environment. However, the simultaneous decline in network complexity and structural stability may increase the risk of abrupt microbiome reorganization. Future research should quantify whether bacterial‐eukaryotic bipartite interactions can offset losses in within domain structural stability. Incorporating cross trophic interaction networks, such as zooplankton‐microbe networks, will be crucial for evaluating multidimensional ecosystem stability under warm‐wet scenarios.

Second, the coordinated changes in functional redundancy index (FRI) and metabolic potential suggest that arid‐region lakes may adapt to climate change partly through functional reorganization rather than taxonomic structure alone. However, the observed decoupling between diversity and community or network structural stability indicates that diversity conservation alone may be insufficient to preserve all dimensions of microbiome stability, even when broad functional potential is maintained. While our mesocosm experiments effectively simulated short‐term microbial responses to future warm‐wet transitions, longer‐term studies encompassing multiple seasons or annual cycles may better capture slower ecological processes and functional turnover. Future research incorporating dynamic mixing conditions and additional ecological parameters (e.g., zooplankton interactions, vertical migrations, and redox sensitive processes) could provide a more holistic understanding of microbial responses to climate change.

## 4 CONCLUSION

This study provides an integrated assessment of how a long‐term warm‐dry to warm‐wet hydroclimatic transition restructures arid lake microbiomes and their functional potential. In Lake Bosten, warm‐wet conditions altered water level, salinity, and nutrient conditions, thereby reshaping bacterial and eukaryotic microbial diversity, community composition, and assembly processes. Both microbial domains showed stronger homogeneous selection and weaker dispersal limitation under warm‐wet conditions, indicating that enhanced hydrological connectivity and more uniform environmental filtering were important mechanisms of community restructuring. However, bacterial and eukaryotic microbes did not respond synchronously, as they differed in taxonomic turnover, diversity responses, and their apparent linkage to nitrogen cycling functions. Warm‐wet conditions reduced within domain community compositional stability and network structural stability, but strengthened bacterial‐eukaryotic bipartite interactions. Despite pronounced taxonomic and network reorganization, broad functional potential was comparatively buffered, likely through functional redundancy, pathway‐specific nitrogen cycling redistribution, and replacement among bacterial functional guilds. These findings highlight that arid lake microbiomes can undergo substantial structural reorganization under warm‐wet hydroclimatic change, while functional potential may be buffered by cross domain coupling and taxonomic compensation.

## 5 METHODS

### Study area and field sampling

Lake Bosten, located in the arid and semi‐arid region of Northwest China, is the largest freshwater inflow–outflow lake in the region. It covers 1064 km^2^ at 1047 m above sea level, with a mean depth of 8 m, a maximum depth of 17 m, and a total water volume of 73 × 10^8^ m^3^ [9, 63]. During summer (July–August), wind‐driven mixing is weak and insufficient to resuspend sediments. The basin experiences strong solar radiation, high evaporation (~1881 mm yr^−1^), and low annual precipitation (64.7 mm), consistent with a temperate continental climate.

Because of its geographic position and hydrological dependence on external inputs, Lake Bosten is highly sensitive to climate variability. From 1958 to 2022, significant warming trends were recorded in the Lake Bosten Basin, as evidenced by air temperature data from the upstream Bayanbulak Station (BYB; elevation 2459 m) and the Yanqi Station (elevation 1057 m) (Figure 2A,B). At Yanqi, the annual mean air temperature increased from 7.5°C in 1958 to 10.2°C in 2022, with an average warming rate of 0.42°C/10a. Although precipitation exhibited an increasing trend overall, spatial variations were evident. In Bayanbulak, annual precipitation rose from 212.2 mm in 1983 to 394.4 mm in 2019, whereas in Yanqi, no significant long‐term increase was observed [9]. These records document a regional transition from a WD to a WW climate regime since the late 1980s, as reported in previous studies [9, 64, 65].

The WW regime has been accompanied by higher temperatures, enhanced glacier melt, and increased precipitation, the main sources of hydrological input to arid‐region lakes. The Kaidu River represents the dominant hydrological input, contributing ~ 95% of total inflow to Lake Bosten [66]. Between 2010 and 2020, glacier area and glacier number in the basin decreased by 36.35 km^2^ and by 175, respectively [67]. Basin‐wide evaporation fluctuated interannually, being markedly higher in 2010−2013 than in 2020−2022 [67].

Anthropogenic influences were also assessed but were minor compared with climate forcing. The Ecological Water Conveyance Project increased outflow during 2003−2012 [66], yet the release volume (e.g., 0.2 × 10^8^ m^3^ in 2010, ~ 0.3% of total lake volume) was negligible relative to natural hydrological fluctuations. Population size and water consumption decreased after 2010 (Figure S20). Consistent with previous studies [9, 68], our earlier analyses showed that WL and TDS changes before 1986 were mainly human induced, whereas variations since 1987 have been primarily climate‐driven [9].

Although the overall regime has remained WW since the late 1980s, a transient WD phase occurred in 2010−2014, reflecting temporary aridification [9, 19]. Based on basin climatic characteristics and data availability, two contrasting climate phases were selected for comparative microbial analysis: the WD period (2010−2011): lower precipitation (52.8 mm), lower air temperature (9.33°C), lower WL (1045.8 m) and higher TDS (1434.1 mg/L); and the WW period (2021−2022): higher precipitation (88.9 mm), higher air temperature (9.78°C), elevated WL (1047.5 m), and reduced TDS (938.0 mg/L).

A mixed spatial‐temporal sampling strategy was implemented to capture major environmental gradients across Lake Bosten. This strategy included matched stations and months across the WD and WW periods, such as stations 1, 2, 4, 7, 10, and 12–16 sampled in July and October of both 2010 and 2021. Additional stations and months, including sites 3, 5, 6, 8, 9, 17, and September samples, were included to expand spatial and temporal coverage. A total of 92 water samples were collected from 17 monitoring sites spanning a 10 year period (Figure S1 and Table S1). This dataset comprises 47 samples from the WD period (2010–2011), and 45 samples from the WW period (2021–2022). Specifically, during the WD period, 47 water samples were collected from 10 monitoring sites (sites 1, 2, 4, 7, 10, and 12–16) between July and October of 2010 and 2011, while during the WW period, 45 samples were collected from 17 sites (sites 1–17) in July, August, and October of 2021 and 2022, covering both consistent and expanded spatial ranges. Each site‐date combination was treated as an independent environmental replicate. This design matched our objective of comparing microbial communities across hydroclimatic phases and lake‐wide environmental gradients. Therefore, the field survey emphasized independent spatial‐temporal replication rather than repeated sequencing of the same DNA extract. At each sampling site, 20 L of surface water was collected, and 800–1000 mL was filtered through 0.2 μm polycarbonate membranes to capture microbial communities. All filters were stored at −80°C and processed together for DNA extraction and sequencing in a single batch to avoid batch effects. Detailed sampling periods and site information are provided in Table S1.

### Mesocosm experiments and sample collection

Field observations indicated that the influence of the warm‐wet climate on the Lake Bosten ecosystem was primarily manifested through two key dimensions: (i) direct temperature effects, and (ii) WL effects, encompassing both the direct role of hydrological connectivity and the indirect role of WL‐driven changes in water chemistry (e.g., salinity and nutrients). Based on 30 years of long‐term monitoring, previous research [9, 19, 25, 28] and projections under future climatic scenarios suggest that water temperature, salinity, and nutrient concentrations (TN and TP) are particularly sensitive to climate forcing. Three considerations were especially important for experimental design. First, analysis of ERA5 and ERA5 Land data, including surface solar radiation downwards, surface net solar radiation, and downward UV radiation, showed no significant differences in light intensity between the WD and WW periods (Figure S18). Therefore, light was not included as a treatment factor in this experiment.

Second, although our field data showed reduced nutrient concentrations under current WW conditions (in contrast to higher levels during WD), mid‐ and long‐term climatic projections suggest that continued warming and increased precipitation in Northwest China could enhance external nutrient inputs into Lake Bosten (a mesotrophic and phosphorus limited lake). Such inputs may counterbalance or even outweigh dilution effects, ultimately leading to elevated nutrient concentrations. Accordingly, nutrient levels under simulated future WD and WW scenarios were designed in the opposite direction to those observed in short‐term field surveys.

Third, Lake Bosten exhibited substantial variability even within the broader warm‐wet regime (Figure 2B,C). Periods of decreased WL and elevated TDS occur intermittently, reflecting short‐term climate fluctuations. To capture this variability, the mesocosm experiment was designed to encompass a broader range of ecological scenarios, extending beyond current field conditions to include projected climatic extremes (e.g., IPCC based scenarios), thereby providing insights into potential microbial responses under diverse future WD and WW conditions. Therefore, the mesocosm experiment was not intended to reproduce the contemporary nutrient contrast observed between the field WD and WW periods, but to explore plausible microbial responses under projected future warm‐dry and warm‐wet hydrochemical scenarios.

To examine potential microbial responses to these projected conditions, we conducted a short‐term mesocosm experiment from July 18 to August 28, 2023, at the Institute of Lake Bosten in Korla, Xinjiang. Surface sediments from Lake Bosten were homogenized and distributed into 18 pre‐sterilized glass tanks (HEK‐600; 69 L; 57.5 × 33.5 × 35.8 cm), each receiving ~ 5 cm of sediment (Figure 1) [25, 28]. Approximately 25 cm of overlying lake water was gently added along the inner tank wall to prevent sediment resuspension. The systems were left undisturbed for 10 days to allow stabilization before treatments were applied [25]. The experimental duration was determined based on our previous Lake Bosten mesocosm studies [25, 28]. The total operation lasted approximately 40 days, including a 10 day stabilization period before treatment application and a 30 day formal treatment period. Previous results showed that microbial communities exhibited strong temporal succession during the first 20 days, whereas diversity patterns and treatment effects became relatively stable by Day 30. Therefore, samples collected on Days 0, 15, and 30 of the formal treatment periods were used to capture the initial condition, intermediate response, and relatively stabilized treatment response to the simulated hydroclimatic scenarios.

The mesocosm experiment comprised nine treatment groups with two replicates per group and was designed as a scenario based 3 × 3 experiment, rather than a complete 3 × 3 × 3 factorial design. Specifically, three temperature levels were crossed with three coupled hydrochemical scenarios: control hydrochemistry, dry hydrochemistry, and wet hydrochemistry (Table S2). Temperature treatments included: ambient (26°C, based on the historical summer mean), moderate warming (+3°C, 29°C), and high warming (+6°C, 32°C) [25, 28], consistent with IPCC projections for the periods 2041−2060 and 2081−2100 under the SSP5‐8.5 scenarios. Water temperature was maintained using variable‐frequency heating devices [25]. The control hydrochemical scenario was defined as a salinity of 1‰ with TN = 0.8 mg/L and TP = 0.01 mg/L. The dry hydrochemical scenario combined increased salinity (1.5‰, Sal+50%) with reduced nutrient concentrations (TN = 0.4 mg/L and TP = 0.005 mg/L, −50%), whereas the wet hydrochemical scenario combined reduced salinity (0.5‰, −50%) with elevated nutrient concentrations (TN = 1.2 mg/L and TP = 0.015 mg/L, +50%). The Sal−50% treatment was prepared by diluting with autoclaved ultrapure water, while the Sal+50% was adjusted using sterilized crystalline salt obtained from the Lake Bosten salt field. The 1‰ threshold approximates the boundary between freshwater and oligo‐saline conditions [9] and reflects alternating dry–wet environmental scenarios. Nutrient concentrations were adjusted using KNO_3_ and K_2_HPO_4_·3H_2_O [69]. Because salinity and nutrient concentrations were intentionally paired to represent ecologically plausible hydrochemical conditions associated with projected dry and wet climate scenarios, they were not independently manipulated as orthogonal factors. Therefore, this design allowed us to compare temperature effects with the combined effects of salinity‐nutrient hydrochemical scenarios, but not to independently partition salinity effects, nutrient effects, or their full factorial interactions with temperature. Overall, T_Warm‐dry1_ and T_Warm‐dry2_ represented future warm‐dry (WD) scenarios, while T_Warm‐wet1_ and T_Warm‐wet2_ simulated future warm‐wet (WW) conditions (Table S2).

Water samples were collected on Days 0, 15, and 30 of the experiment. For each sampling event, 800 mL of mixed water was collected from each tank. In total, 54 samples were obtained. Samples were filtered through 0.2 μm polycarbonate membranes to capture microbes, and the filters were stored at −80°C for subsequent DNA extraction.

### DNA extraction, amplicon sequencing, and analysis

Microbial DNA was extracted from the polycarbonate filters using the Soil SPINeasy^TM^ DNA Pro Kit (MP Biomedicals, USA), following the manufacturer's instructions. For bacterial community analysis, the V3–V4 region of the 16S rRNA gene was amplified using the primer pair 341F (5′‐CCTACGGGNGGCWGCAG‐3′) and 806R (5′‐GGACTACHVGGGTATCTAAT‐3′). For eukaryotic microbial communities, the V4 region of the 18S rRNA gene was amplified using the primer pair 528F (5′‐GCGGTAATTCCAGCTCCAA‐3′) and 706R (5′‐AATCCRAGAATTTCACCTCT‐3′). Polymerase chain reaction (PCR) amplification was performed according to a previously described protocol [70]. The amplified DNA fragments were sequenced with 2 × 250 bp paired‐end reads on an Illumina MiSeq platform (Guangzhou Gene Denovo Biotechnology Co., Ltd.) following standard sequencing protocols. To monitor potential contamination during filtration, DNA extraction, and PCR amplification, negative controls were included throughout the workflow. Sterilized Milli‐Q water was filtered under the same conditions as the field water samples and then subjected to the same DNA extraction and PCR amplification procedures. No visible PCR products were detected from these negative controls, indicating that contamination was not introduced during sample processing or amplification.

Bioinformatic analyses were conducted using QIIME2, with the DADA2 plugin [71] for sequence trimming, chimera removal, denoising, and quality control [72]. This workflow generated a feature table containing amplicon sequence variants (ASVs), which were subsequently assigned taxonomic identities using a Naive Bayes classifier referencing the SILVA 138 database [73]. For bacterial community analyses, sequences assigned to archaea, chloroplasts, mitochondria, and unassigned taxa were excluded. Similarly, for eukaryotic communities, sequences classified as archaea, bacteria, metazoa, arthropoda, annelida, aphelidea, holozoa, mollusca, nemertea, rotifera, vertebrata, embryophytes, or unassigned taxa were removed. To minimize the impact of random sequencing errors, ASVs with fewer than 10 reads were filtered out [19, 25]. Sequencing sufficiency was further evaluated using rarefaction curves for bacterial and eukaryotic communities, which approached clear plateaus and indicated that the retained sequencing depth was sufficient for downstream diversity analyses.

### Metagenome sequencing and analysis

A subset of 28 samples, comprising 10 from field sampling and 18 from mesocosm experiments, was selected for metagenomic sequencing to investigate microbial functional characteristics. Metagenomic libraries were constructed using the ALFA‐SEQ DNA Library Prep Kit (Findrop, Guangzhou) following the manufacturer's protocol. Sequencing was performed on the Illumina NovaSeq 6000 platform by Guangdong Magigene Biotechnology Co., Ltd., generating paired‐end reads of 150 bp. In total, 2.43 × 10^9^ raw reads were obtained, yielding 364.6 Gb of sequencing data, with an average of 13 Gb per sample (ranging from 10.7 to 15.9 Gb). To ensure high data quality and reliability, raw sequencing reads were processed using Fastp (v0.21.0) with the following parameters: ‘−5 ‐W 5 ‐M 20 ‐q 15 ‐u 40 ‐ 50 –dedup’ [74]. The quality control steps included: removal of adapter sequences, elimination of low quality reads (quality score < 15), removal of short reads (< 50 bp), and deduplication of PCR amplified sequences. Sequencing quality was verified using FastQC (v0.11.9) [58, 74]. Clean reads were then assembled de novo using MEGAHIT (v1.2.9) [75] with k‐mer values set to k‐min 35, k‐max 95, and k‐step 20. Contigs ≥ 500 bp were used for further analysis, and the assembly quality was evaluated based on N50. ORF prediction was performed on contigs (≥ 500 bp) using Prodigal (v2.6.3) [76], with ORFs < 90 bp discarded. Clean reads were mapped to predicted ORFs via BBMap, retaining only ORFs with ≥ 1 mapped read and lengths matching the predicted ORF length. Non‐redundant gene catalogues were generated using Linclust [77], selecting the longest sequence per cluster as the representative Unigenes. Clean reads were re‐mapped to the Unigene catalogue using BBMap to calculate read counts and average coverage depth. Gene relative abundance was determined using:

$$G_{k}=\frac{r_{k}}{L_{K}}\times \frac{1}{\sum_{i}^{n}\frac{r_{i}}{L_{i}}},$$

where *i* denotes a gene, *n* is the total number of genes, *r*_*k*_ is the number of reads mapped to gene *k*, and *L*_*k*_ is the length of gene *k*. The non‐redundant gene catalogue was functionally annotated using DIAMOND (v2.1.6) [78] against the KEGG [79] and eggNOG (v6.0) [80] databases (e value ≤ 0.001).

### Microbial community and network stability analysis

Stability is multidimensional, and different metrics capture different ecological properties. In this study, we explicitly distinguished community compositional stability from network structural stability. At the community level, stability was quantified using the AVD index [28, 81], which measures the fluctuation of microbial relative abundance across samples. A lower AVD value indicates a more stable community, as it reflects reduced variability in species abundance among samples within a given period. The AVD values were calculated using the following equation:

$$\text{AVD}=\frac{\sum_{i=1}^{n}\frac{\left|x_{i}-{\bar{x}}_{i}\right|}{\delta_{i}}}{k\times n},$$

where $x_{i}\;$denotes the ASV abundance in a given sample, ${\bar{x}}_{i}\;$represents the mean ASV abundance within a sample group, $\delta_{i}\;$is the standard deviation of ASV abundances within that group, k indicates the total number of samples in the group, and n refers to the number of ASVs present in each group.

At the network level, network structural stability was evaluated using Robustness and Vulnerability metrics [25, 29, 31, 44, 82]. Robustness reflects the percentage of species that persist in the network after either random or targeted node removal [29]. To examine the effects of species loss on microbial network stability, we conducted 100 simulations in which 50% of the nodes were randomly eliminated [25, 29]. Vulnerability reflects the degree to which a network is affected by the loss of individual nodes and was calculated using [28, 29]:

$$\max(\frac{E-E_{i}}{E}),$$

where *E* and *E*_*i*_ are the global efficiency when node *i* and its edges are removed. Global efficiency was computed as:

$$E=\frac{1}{n(n-1)}\sum_{i\neq j}\frac{1}{d(i,j)},$$

where *d* (*i*, *j*) represents the number of edges in the shortest path between node *i* and *j*. Higher vulnerability indicates reduced stability.

### Statistical analyses

A comprehensive suite of statistical analyses was performed in R (v4.4.2) to investigate the diversity, stability, assembly processes, functional traits, and underlying driving mechanisms of aquatic microorganisms under climate warm‐dry and warm‐wet scenarios. Long‐term hydrometeorological trends in the Lake Bosten basin were assessed using the modified non‐parametric Mann–Kendall test, implemented via the “modifiedmk” package v1.5.0 [9]. Aquatic environmental heterogeneity was quantified based on eight key parameters, following previously established protocols (Supplementary Information). Differences in environmental variables and microbial diversity between the WD and WW periods were assessed using Wilcoxon rank sum tests. For families of related comparisons, including environmental variables and alpha diversity indices, *p* values were adjusted using the Benjamini–Hochberg false discovery rate (FDR) procedure, with *q* < 0.05 considered statistically significant. To infer microbial community assembly mechanisms between the WD and WW periods, we applied the iCAMP framework [83], as detailed in the Supplementary Information.

To visualize differences in microbial community structure and function, PCoA based on Bray–Curtis distances was performed, and differences between groups were tested using PERMANOVA with 999 permutations. For the marginal boxplots in the PCoA plots, differences in PCoA1 and PCoA2 scores between periods were evaluated using one‐way ANOVA followed by Tukey multiple comparisons (*α* = 0.05). To further elucidate the mechanisms underlying changes in microbial communities and functions under warm‐wet climate, RDA was conducted using environmental parameters and ASV or functional compositions (KEGG and COG module levels). Before RDA, ASV, and functional tables were transformed using the Hellinger method, and Monte Carlo permutation tests with 999 permutations were used to select significant environmental variables. Variables with variance inflation factors (VIF > 10) [19] or non‐significant contributions (*p* > 0.05) were excluded, and their contributions quantified using the “rdacca.hp” package [84].

Because the field survey included both matched and additional stations and months, we performed a matched site × month sensitivity analysis to evaluate whether the main patterns from WD to WW were robust to unequal spatial and temporal coverage. Only site × month combinations present in both WD and WW periods were retained (25 pairs). When multiple samples occurred within the same type × site × month combination, values were averaged before paired comparison. Alpha diversity metrics and environmental variables were tested using paired *t* tests and Wilcoxon signed rank tests. Community composition was tested using matched‐blocked PERMANOVA based on Bray–Curtis dissimilarities of ASV relative abundances, with site × month pairs treated as blocking units. The matched analysis supported the full dataset results, confirming that the observed differences between WD and WW were not driven by treating all field samples as fully independent (Figure S21).

Differential abundance analysis was conducted using “DESeq2” [85] to calculate log_2_‐fold changes in ASV abundance between the WW and WD periods. *p* values from “DESeq2” were adjusted using the Benjamini–Hochberg FDR procedure, and ASVs with *q* < 0.05 were considered significantly responsive to the climate period. Associations between responsive ASVs and physicochemical variables were evaluated using Pearson correlation analysis. For these ASV‐environment correlation tests, *p* values were also adjusted using the Benjamini‐Hochberg FDR procedure, and *q* < 0.05 was used as the significance threshold. Representative ASV sequences with significant differences were aligned using the MUSCLE [86] algorithm with default parameters, and phylogenetic trees were constructed using FastTree [87] with the Maximum Likelihood (ML) method. Visualization of phylogenetic results was generated using the “itol.toolkit” [88] in R and the interactive tree of life (iTOL) platform [89]. For microbial functional gene analysis, KEGG annotations were used to identify genes and pathways related to carbon, nitrogen, phosphorus, and sulfur cycling. Log_2_‐fold changes were quantified via the “edgeR” [90] package, and functional redundancy was estimated with Tax4Fun2 [91]. For gene differential abundance analyses, *p* values were adjusted using the Benjamini–Hochberg FDR procedure, and *q* < 0.05 was used as the significance threshold. The FRI was computed to indicate the distribution of functions among community members.

To identify environmental drivers of microbial diversity, LMMs were fitted using the “lme4” [92] package in R. Candidate predictor sets were constructed using three complementary sources: ecological prior knowledge from published literature, variable importance from random forest analysis, and significant explanatory variables identified by RDA after collinearity screening. This multi‐source strategy was used to define candidate models rather than to independently interpret all selected variables. Candidate LMMs were then compared using Akaike information criterion, Bayesian information criterion, and log‐likelihood values, and inference was based on the best model. Sampling site was included as a random effect where appropriate to reduce the influence of spatial non‐independence. Effect sizes were represented by standardized regression coefficients derived from *z*‐transformed variables, rendering them unitless and directly comparable across predictors. Model significance was assessed using ANOVA from the “car” package, and the relative contributions of predictors in the final selected model were quantified using the “glmm.hp” [93] package in R. Details on variable selection and comparisons among candidate model formulations are provided in the Supplementary Information.

Finally, SEM was performed using the “lavaan” package [94] as an exploratory path analysis to summarize hypothesized associations among climate conditions, environmental parameters, microbial diversity, community compositional stability, functional redundancy, and predicted functional traits. Model fit was assessed using four key indices: Chi‐squared (*χ* ^2^) test, root mean square error of approximation (RMSEA), comparative fit index (CFI), and normed fit index (NFI) [27, 58]. Specifically, better model performance was indicated by CFI and NFI values approaching 1, an RMSEA value close to 0, and higher χ^2^ and RMSEA *p* values. Path coefficients and associated *p* values were interpreted to infer the strength and direction of relationships between variables. In the SEM, climate conditions (temperature and precipitation) represented the transition from WD to WW; microbial diversity was represented by PCA1 of the Shannon index, Observed ASVs, and Faith's PD; microbial functional traits in the SEM were represented by PCoA1 of FAPROTAX‐predicted functional profiles derived from bacterial 16S rRNA gene data; community compositional stability was represented by 1‐AVD; and functional redundancy by FRI.

## AUTHOR CONTRIBUTIONS

**Zhen Shen**: investigation, formal analysis, methodology, validation, visualization, writing − original draft, writing − review and editing. **Xingchen Li**: formal analysis, methodology, software, validation; visualization. **Zhijun Gong**: methodology; validation, resources, supervision, writing − review and editing. **Yang Hu**: investigation, data curation, project administration, writing − review and editing. **Yongjiu Cai**: data curation, supervision, writing − review and editing. **Keqiang Shao**: investigation, project administration, supervision, writing − review and editing. **Guang Gao**: conceptualization, investigation, funding acquisition, methodology, writing − review and editing. **Robbie M. Martin**: writing − review and editing. **Xiangming Tang**: conceptualization, investigation, methodology, data curation, formal analysis, writing − review and editing.

## CONFLICT OF INTEREST STATEMENT

The authors declare no conflicts of interest.

## ETHICS STATEMENT

No animals or humans were involved in this study.

## Supporting information


**Figure S1:** Seventeen monitoring points in Lake Bosten.
**Figure S2:** Spearman correlation of water physicochemical parameters in warm‐dry and warm‐wet periods.
**Figure S3:** Water environmental parameters and environmental heterogeneity during two contrasting periods: warm‐dry and warm‐wet.
**Figure S4:** Faith's PD diversity across warm‐dry and warm‐wet periods.
**Figure S5:** Effects of environmental parameters on microbial diversity, represented as regression coefficients from linear mixed effects models.
**Figure S6:** Bacterial and eukaryotic microbial diversity across scenario based mesocosm treatments.
**Figure S7:** Principal Coordinate Analysis (PCoA) of bacterial and eukaryotic community structures based on Bray‐Curtis distance.
**Figure S8:** Bacterial and eukaryotic community composition at the genus level during warm‐dry and warm‐wet periods.
**Figure S9:** Redundancy analysis (RDA) of environmental drivers of microbial community structure and functional composition.
**Figure S10:** *Z_i_*‐*P_i_* plot categorizing ASVs based on their topological roles within bacterial networks.
**Figure S11:** Topological roles of nodes in bacterial‐eukaryotic bipartite networks, identifying module hubs, connectors, and network hubs as keystone nodes.
**Figure S12:** Functional gene composition during warm‐dry and warm‐wet periods in field surveys and mesocosm experiments.
**Figure S13:** Microbial functional composition during field surveys and mesocosm experiments.
**Figure S14:** Dominant taxonomic contributors to nitrogen cycling functions during the warm‐dry (WD) and warm‐wet (WW) periods.
**Figure S15:** Triangular plots of β diversity patterns based on Jaccard dissimilarity for eukaryotic microbial communities across warm‐dry and warm‐wet periods.
**Figure S16:** Relative sequence abundance of autotrophic photosynthetic microorganisms during warm‐dry and warm‐wet periods.
**Figure S17:** Relative abundance of photosynthesis related functions during warm‐dry and warm‐wet periods.
**Figure S18:** Light environment during warm‐dry and warm‐wet periods at Lake Bosten.
**Figure S19:** Functional redundancy was estimated using Tax4Fun2, which predicts functional phylogenetic distributions based on KEGG annotations.
**Figure S20:** Long‐term changes in population and water consumption in the Kaidu River catchment during 1958−2019.
**Figure S21:** Matched site × month sensitivity analyses of microbial diversity, community composition, and environmental shifts between warm‐dry and warm‐wet periods.

**Table S1:** Sampling site details for warm‐dry (WD) and warm‐wet (WW) periods.
**Table S2:** Scenario based mesocosm treatment design crossing three temperature levels with three coupled hydrochemical scenarios.
**Table S3:** Range analysis of bacterial diversity responses across temperature levels and coupled hydrochemical scenarios in the mesocosm experiment.
**Table S4:** Range analysis of eukaryotic microbial diversity responses across temperature levels and coupled hydrochemical scenarios in the mesocosm experiment.
**Table S5:** Topological properties of bacterial and eukaryotic networks during warm‐dry (WD) and warm‐wet (WW) periods.
**Table S6:** Keystone information of bacterial networks during warm‐dry (WD) and warm‐wet (WW) periods.
**Table S7:** Keystone taxa and putative ecological functions in the bacterial‐eukaryotic dual network during warm‐dry (WD) and warm‐wet (WW) periods.
**Table S8:** Abundance of nitrogen cycling marker genes expressed as TPM (Transcripts Per Million) during warm‐dry (WD) and warm‐wet (WW) periods.

## Data Availability

All data required to reproduce the results are available in the Figshare Database (https://doi.org/10.6084/m9.figshare.29133269). Raw sequencing data have been deposited in the Genome Sequence Archive (GSA) at the National Genomics Data Center (NGDC), China National Center for Bioinformation/Beijing Institute of Genomics, Chinese Academy of Sciences, under accession numbers CRA022921 (bacterial sequences: https://ngdc.cncb.ac.cn/gsa/browse/CRA022921) and CRA013104 (eukaryotic sequences: https://ngdc.cncb.ac.cn/gsa/browse/CRA013104). The ERA5 Land data used in this study are available at https://cds.climate.copernicus.eu/datasets/reanalysis-era5-land?tab=overview. The ERA5 hourly data for downward UV radiation are available at: https://cds.climate.copernicus.eu/datasets/reanalysis-era5-single-levels. Source data supporting the findings of this study are provided with the paper. The analysis code is available at GitHub (https://github.com/zhenshen0830/Warm-wet-climate-impacts-on-lake-microbes). Supplementary materials (methods, figures, tables, graphical abstract, slides, videos, Chinese translated version, and updated materials) may be found in the online DOI or iMeta Science http://www.imeta.science/imetaomics/.
