# Supplementary material for: Warm‐wet transition restructures arid lake microbiomes and functional potential
Source: IMetaOmics. 2026 Jul 8;3(3):e70119. doi: 10.1002/imo2.70119 (PMC13674413; doi:10.1002/imo2.70119)
Supplement: Supplementary file 1 [file IMO2-3-e70119-s001.docx]

Supporting information to

**Warm-wet transition restructures arid lake microbiomes and functional potential**

**Running title:** Warm-wet climate reshapes arid lake microbes

Zhen Shen^1,2^, Xingchen Li^3^, Zhijun Gong^1,2*^, Yang Hu^1^, Yongjiu Cai^1,2^, Keqiang Shao^1,2^, Guang Gao^1,2^, Robbie M. Martin^4^, Xiangming Tang^1,2*^

^1^State Key Laboratory of Lake and Watershed Science for Water Security, Nanjing Institute of Geography and Limnology, Chinese Academy of Sciences, Nanjing 211135, China

^2^University of Chinese Academy of Sciences, Beijing 100049, China

^3^School of Earth and Environment, Anhui University of Science & Technology, Huainan 232001, China

^4^Department of Microbiology, University of Tennessee, Knoxville, Tennessee 37996, United States

*Correspondence: zjgong@niglas.ac.cn (Zhijun Gong); [xmtang@niglas.ac.cn](mailto:xmtang@niglas.ac.cn) (Xiangming Tang)

This supplementary information contains:

Supplementary Materials and Methods

Supplementary Figures S1 to S21

Supplementary References

**METHODS**

**Environmental parameter analysis**

At each sampling site, pH, electrical conductivity (EC), dissolved oxygen (DO), salinity (Sal), total dissolved solid (TDS), and water temperature (WT) were measured in the field using multi-parameter water quality sonde (YSI 6600v2, USA). Transparency (SD) was measured with a Secchi disk. Water depth (WD) was measured by a depth sounder. An additional 5 environmental parameters including: chemical oxygen demand (COD), total nitrogen (TN), total suspended solids (TSS), chlorophyll-*a* (Chl.*a*), total phosphorus (TP), were analyzed using standard methods [1].

**Network construction**

Microbial networks were constructed using Spearman’s correlation coefficients, computed via the Integrated Network Analysis Pipeline (iNAP, <https://inap.denglab.org.cn/>) [2]. To ensure robust correlations, only ASVs present in at least half of the total samples were included in the network analysis. Prior to calculations, zero and empty values were substituted with a small constant (e.g., 0.01) to enhance reliability [2]. Networks were constructed using similarity thresholds of 0.81 for bacteria and 0.7 for eukaryotes, as determined by random matrix theory (RMT) [3]. Key network properties—including the total number of nodes and links, average clustering coefficient (AvgCC), average path length (GD), average degree (AvgK), harmonic geodesic distance (HD), and geodesic efficiency (GE), were computed using the “MEN analysis” module in iNAP. Additionally, 100 randomized networks were generated by rewiring nodes and edges in each empirical network. Paired Student’s *t*-tests were performed to compare structural differences between empirical and random networks, with mean and standard variance values reported [2]. Network visualizations were generated using Cytoscape (v3.9.1).

**Network complexity analysis**

To quantify complexity within network analysis, various topological properties are assessed, such as the number of nodes, links, AvgK, GE, connectance, and density [2,4,5]. To provide a more comprehensive measure of microbial network complexity, a composite index was developed. This index was calculated as the mean of standardized values for multiple key network properties: the number of nodes, links, AvgK, AvgCC, GD, GE, centralization of betweenness, centralization of stress centrality, density, and connectedness. The final formula for this complexity index is:

$$Network complexity=\frac{(X_{raw}-X_{min})}{(X_{max}-X_{min})}$$

where *X_raw_*, *X_min_*, *X_max_* represent the raw topological properties, and their minimum and maximum values across all plots, respectively.

**Linear mixed-effects models analysis**

To ensure accurate model selection, we identified influencing factors using three approaches:

1. Based on published literature, we selected five key factors:

Bacteria: Water Level (WL), TDS, WT, TN, and Chl.*a*

(1) Observed ASVs

mod1: Observed ASVs ~ TN + WT + Chl.*a* + TDS + WL + (1 | site)

mod2: Observed ASVs ~ TN + WT + Chl.*a* + TDS + WL + (0 + Type | site)

mod3: Observed ASVs ~ TN + WT + Chl.a + TDS + WL + (Type | site)

mod4: Observed ASVs ~ TN + WT + Chl.a + TDS + WL + (Type || site)

(2) Shannon

mod1: Shannon ~ TN + WT + Chl.a + TDS + WL + (1 | site)

mod2: Shannon ~ TN + WT + Chl.a + TDS + WL + (0 + Type | site)

mod3: Shannon ~ TN + WT + Chl.a + TDS + WL + (Type | site)

mod4: Shannon ~ TN + WT + Chl.a + TDS + WL + (Type || site)

(3) Faith PD

mod1: Faith PD ~ TN + WT + Chl.a + TDS + WL + (1 | site)

mod2: Faith PD ~ TN + WT + Chl.a + TDS + WL + (0 + Type | site)

mod3: Faith PD ~ TN + WT + Chl.a + TDS + WL + (Type | site)

mod4: Faith PD ~ TN + WT + Chl.a + TDS + WL + (Type || site)

Eukaryotic microorganisms: WL, TN, TDS, Chl.*a*, WT

(4) Observed ASVs

mod1: Observed ASVs ~ TN + WT + Chl.a + TDS + WL + (1 | site)

mod2: Observed ASVs ~ TN + WT + Chl.a + TDS + WL + (0 + Type | site)

mod3: Observed ASVs ~ TN + WT + Chl.a + TDS + WL + (Type | site)

mod4: Observed ASVs ~ TN + WT + Chl.a + TDS + WL + (Type || site)

(5) Shannon

mod1: Shannon ~ TN + WT + Chl.a + TDS + WL + (1 | site)

mod2: Shannon ~ TN + WT + Chl.a + TDS + WL + (0 + Type | site)

mod3: Shannon ~ TN + WT + Chl.a + TDS + WL + (Type | site)

mod4: Shannon ~ TN + WT + Chl.a + TDS + WL + (Type || site)

(6) Faith PD

mod1: Faith PD ~ TN + WT + Chl.a + TDS + WL + (1 | site)

mod2: Faith PD ~ TN + WT + Chl.a + TDS + WL + (0 + Type | site)

mod3: Faith PD ~ TN + WT + Chl.a + TDS + WL + (Type | site)

mod4: Faith PD ~ TN + WT + Chl.a + TDS + WL + (Type || site)

2. Using a random forest model implemented through the ‘randomForest’ and ‘rfPermute’ packages in R, we selected five factors:

Bacteria: TDS, TN, pH, WL, DO

(7) Observed ASVs

mod1: Observed ASVs ~ TN + TDS + WL + pH + DO + (1 | Site)

mod2: Observed ASVs ~ TN + TDS + WL + pH + DO + (0 + Type | Site)

mod3: Observed ASVs ~ TN + TDS + WL + pH + DO + (Type | Site)

mod4: Observed ASVs ~ TN + TDS + WL + pH + DO + (Type || Site)

(8) Shannon

mod1: shannon ~ TN + DO+ pH + TDS + WL + (1 | Site)

mod2: shannon ~ TN + DO+ pH + TDS + WL + (0 + Type | Site)

mod3: shannon ~ TN + DO+ pH + TDS + WL + (Type | Site)

mod4: shannon ~ TN + DO+ pH + TDS + WL + (Type || Site)

(9) Faith PD

mod1: Faith PD ~ TN + DO+ pH + TDS + WL + (1 | Site)

mod2: Faith PD ~ TN + DO+ pH + TDS + WL + (0 + Type | Site)

mod3: Faith PD ~ TN + DO+ pH + TDS + WL + (Type | Site)

mod4: Faith PD ~ TN + DO+ pH + TDS + WL + (Type || Site)

Eukaryotic microorganisms: TN, WT, TDS, WL, pH

(10) Observed ASVs

mod1: Observed ASVs ~ TN + WT + pH + TDS + WL + (1| site)

mod2: Observed ASVs ~ TN + WT + pH + TDS + WL + (0 + Type | site)

mod3: Observed ASVs ~ TN + WT + pH + TDS + WL + (Type | site)

mod4: Observed ASVs ~ TN + WT + pH + TDS + WL + (Type || site)

(11) Shannon

mod1: Shannon ~ TN + WT + pH + TDS + WL + (1| site)

mod2: Shannon ~ TN + WT + pH + TDS + WL + (0 + Type | site)

mod3: Shannon ~ TN + WT + pH + TDS + WL + (Type | site)

mod4: Shannon ~ TN + WT + pH + TDS + WL + (Type || site)

(12) Faith PD

mod1: Faith PD ~ TN + WT + pH + TDS + WL + (1| site)

mod2: Faith PD ~ TN + WT + pH + TDS + WL + (0 + Type | site)

mod3: Faith PD ~ TN + WT + pH + TDS + WL + (Type | site)

mod4: Faith PD ~ TN + WT + pH + TDS + WL + (Type || site)

3. Based on results from Redundancy Analysis (RDA), we selected five factors:

Bacteria: WL, TDS, DO, SSRDS, TN

(13) Observed ASVs

mod1: Observed ASVs ~ TN + DO + TDS + SSRDS + WL + (1 | Site)

mod2: Observed ASVs ~ TN + DO + TDS + SSRDS + WL + (0 + Type | Site)

mod4: Observed ASVs ~ TN + DO + TDS + SSRDS + WL + (Type || Site)

mod3: Observed ASVs ~ TN + DO + TDS + SSRDS + WL + (Type | Site)

(14) Shannon

mod1: shannon ~ TN + DO + TDS + SSRDS + WL + (1 | Site)

mod2: shannon ~ TN + DO + TDS + SSRDS + WL + (0 + Type | Site)

mod4: shannon ~ TN + DO + TDS + SSRDS + WL + (Type || Site)

mod3: shannon ~ TN + DO + TDS + SSRDS + WL + (Type | Site)

(15) Faith PD

mod1: Faith PD ~ TN + DO + TDS + SSRDS + WL + (1 | Site)

mod2: Faith PD ~ TN + DO + TDS + SSRDS + WL + (0 + Type | Site)

mod4: Faith PD ~ TN + DO + TDS + SSRDS + WL + (Type || Site)

mod3: Faith PD ~ TN + DO + TDS + SSRDS + WL + (Type | Site)

Eukaryotic microorganisms: TN, WT, Chl.*a*, TDS, WL, DO

(16) Observed ASVs

mod1: Observed ASVs ~ TN + WT + Chl.a + TDS + WL + DO + (1 | site)

mod2: Observed ASVs ~ TN + WT + Chl.a + TDS + WL + DO + (0 + Type | site)

mod3: Observed ASVs ~ TN + WT + Chl.a + TDS + WL + DO + (Type | site)

mod4: Observed ASVs ~ TN + WT + Chl.a + TDS + WL + DO + (Type || site)

(17) Shannon

mod1: Shannon ~ TN + WT + Chl.a + TDS + WL + DO + (1 | site)

mod2: Shannon ~ TN + WT + Chl.a + TDS + WL + DO + (0 + Type | site)

mod3: Shannon ~ TN + WT + Chl.a + TDS + WL + DO + (Type | site)

mod4: Shannon ~ TN + WT + Chl.a + TDS + WL + DO + (Type || site)

(18) Faith PD

mod1: Faith PD ~ TN + WT + Chl.a + TDS + WL + DO + (1 | site)

mod2: Faith PD ~ TN + WT + Chl.a + TDS + WL + DO + (0 + Type | site)

mod3: Faith PD ~ TN + WT + Chl.a + TDS + WL + DO + (Type | site)

mod4: Faith PD ~ TN + WT + Chl.a + TDS + WL + DO + (Type || site)

The best model was determined based on comparisons of Akaike Information Criterion (AIC), Bayesian Information Criterion (BIC), and log-likelihood (logLik) values.

**Microbial assembly mechanisms analysis**

To quantify microbial community assembly mechanisms during warm-dry and warm-wet periods, we used a phylogenetic bin-based null model framework implemented in the iCAMP (Infer Community Assembly Mechanisms by Phylogenetic-bin-based null model) R package [6]. Unlike conventional approaches that directly use OTU or ASV data, iCAMP first classifies detected OTUs or ASVs into phylogenetic groups based on their evolutionary relationships and subsequently determines the ecological processes governing each group [7]. The inferred processes include dispersal-related mechanisms, such as homogeneous dispersal (HD) and dispersal limitation (DL), as well as selection-driven processes, including homogeneous selection (HoS) and heterogeneous selection (HeS). Additionally, stochastic processes such as ecological drift (DR) are accounted for, capturing random fluctuations in microbial community composition [6]. In this study, the phylogenetic signal threshold (d_S_) was set to an empirical value of 0.2 to ensure an accurate classification of microbial taxa into distinct phylogenetic groups. The minimum sample size (*n*_min_) was determined as 48 by evaluating a range of *n*_min_ values to optimize the reliability of assembly process quantification.

**Environmental** **heterogeneity assessment**

Environmental heterogeneity (EH) was estimated by calculating the dissimilarity between sampling sites [7,8]. We selected 8 environmental parameters for this analysis: EC, pH, DO, TDS, WT, TN, COD, and Chl.*a*. These parameters were first standardized using z-scores to ensure comparability. Next, the Euclidean distance matrix was calculated in R, which provides a measure of dissimilarity between each pair of sampling sites. The EH was then calculated using the following equation [7,8]:

Eh =$\frac{\mathrm{Euc}}{\mathrm{Euc}_{\max}}+0.001$

where Euc represents the Euclidean distance between two sites, and Euc_max_ denotes the maximum Euclidean distance observed among all site pairs. The constant 0.001 was added to account for zero similarity between sites, ensuring that the calculated Eh is not zero.

**REFERENCES**

1.	Jin, Xiangcan, and Qingying Tu. 1990. The standard methods for observation and analysis in lake eutrophication, 2nd ed. Chinese Environmental Science Press, Beijing.

2.	Feng, Kai, Xi Peng, Zheng Zhang, Songsong Gu, Qing He, Wenli Shen, Zhu Jun Wang, et al. 2022. “iNAP: An integrated network analysis pipeline for microbiome studies.” *iMeta* 1: e13. https://doi.org/10.1002/imt2.13

3.	Deng, Ye, Yihuei Jiang, Yunfeng Yang, Zhili He, Feng Luo, and Jizhong Zhou. 2012. “Molecular ecological network analyses.” *BMC Bioinformatics* 13: 1–20. https://doi.org/10.1186/1471-2105-13-113

4.	Shen, Zhen, Guijuan Xie, Bobing Yu, Yuqing Zhang, Keqiang Shao, Yi Gong, Guang Gao, and Xiangming Tang. 2024. “Eutrophication diminishes bacterioplankton functional dissimilarity and network complexity while enhancing stability: Implications for the management of eutrophic lakes.” *Journal of Environmental Management* 352: 120119. https://doi.org/10.1016/j.jenvman.2024.120119

5.	Shen, Zhen, Bobing Yu, Keqiang Shao, Guang Gao, and Xiangming Tang. 2023. “Warming reduces microeukaryotic diversity, network complexity and stability.” *Environmental Research* 238: 117235. https://doi.org/10.1016/j.envres.2023.117235

6.	Ning, Daliang, Mengting Yuan, Linwei Wu, Ya Zhang, Xue Guo, Xishu Zhou, Yunfeng Yang, Adam P. Arkin, Mary K. Firestone, and Ji Zhong Zhou. 2020. “A quantitative framework reveals ecological drivers of grassland microbial community assembly in response to warming.” *Nature Communications* 11: 4717. https://doi.org/10.1038/s41467-020-18560-z

7.	Shen, Zhen, Bobing Yu, Yi Gong, Keqiang Shao, Guang Gao, and Xiangming Tang. 2024. “Unraveling the impact of climatic warming and wetting on eukaryotic microbial diversity and assembly mechanisms: A 10-year case study in Lake Bosten, NW China.” *Water Research* 256: 121559. https://doi.org/10.1016/j.watres.2024.121559

8.	Huber, Paula, Sebastian Metz, Fernando Unrein, Gisela Mayora, Hugo Sarmento, and Melina Devercelli. 2020. “Environmental heterogeneity determines the ecological processes that govern bacterial metacommunity assembly in a floodplain river system.” *The ISME Journal* 14: 2951–2966. https://doi.org/10.1038/s41396-020-0723-2

# Figure S1 Seventeen monitoring points in Lake Bosten. The sampling strategy incorporated both: (1) Matched stations and months across WD and WW periods (e.g., stations 1, 2, 4, 7, 10, and 12–16 sampled in July and October of both 2010 and 2021); and (2) Additional stations and months (e.g., sites 3, 5, 6, 8, 9, 17 and the month of September) to expand spatial and temporal coverage. This mixed design was intended to capture broad environmental gradients, enhance ecological representativeness, and support robust statistical comparisons within and across periods. A total of 92 water samples were collected systematically from 17 monitoring sites spanning a 10-year period.

# Figure S2 Spearman correlation of water physicochemical parameters in warm-dry and warm-wet periods. The size of each box is scaled according to the absolute value of the Spearman correlation coefficient (|*r*|); larger boxes represent stronger correlations. Numbers within boxes indicate Spearman correlation coefficients. *p* values were adjusted using the Benjamini-Hochberg false discovery rate (FDR) procedure. Electrical conductivity (EC), pH, dissolved oxygen (DO), total dissolved solids (TDS), water temperature (WT), total nitrogen (TN), chemical oxygen demand (COD), and chlorophyll-*a* (Chl.*a*), Transparency (SD), Water depth (WD). Significance: ****q* < 0.001, ***q* < 0.01, **q* < 0.05.

# Figure S3 Water environmental parameters and environmental heterogeneity during two contrasting periods: warm-dry and warm-wet. Each point represents a single water sample collected at a specific lake site and date, either during the warm-dry period (2010–2011, *n* = 47) or the warm-wet period (2021–2022, *n* = 45). Environmental heterogeneity (EH) was quantified using eight parameters: electrical conductivity (EC), pH, dissolved oxygen (DO), total dissolved solids (TDS), water temperature (WT), total nitrogen (TN), chemical oxygen demand (COD), and chlorophyll-a (Chl.*a*). Significant differences between periods were assessed using Wilcoxon rank-sum tests, and *p* values were adjusted across environmental variables using the Benjamini-Hochberg false discovery rate (FDR) procedure. Different lowercase letters indicate significant differences between periods at *q* < 0.05; variables without letter annotations showed no significant differences after FDR correction. Water level (WL); transparency (SD); dissolved oxygen (DO); total suspended solids (TSS); water depth (WD).

# Figure S4 Faith’s PD diversity across warm-dry and warm-wet periods. Each point represents microbial diversity metrics from a single water sample collected at a specific lake site and date, either during the warm-dry period (2010–2011, *n* = 47) or the warm-wet period (2021–2022, *n* = 45).

# Figure S5 Effects of environmental parameters on microbial diversity, represented as regression coefficients (mean ± s.e.m.) from linear mixed-effects models (*n* = 92). Positive coefficients indicate a positive association, whereas negative coefficients reflect suppressive effects on diversity. Significance: ****p* < 0.001, ***p* < 0.01, **p* < 0.05.

# Figure S6 Bacterial and eukaryotic microbial diversity across scenario-based mesocosm treatments. (A–C) Bacterial diversity across the nine mesocosm treatments, represented by Observed ASVs, Shannon index, and Faith’s phylogenetic diversity (PD), respectively. (D–F) Eukaryotic microbial diversity across the nine mesocosm treatments, represented by Observed ASVs, Shannon index, and Faith’s PD, respectively. The nine treatments were generated by crossing three temperature levels with three coupled hydrochemical scenarios: control, dry-associated, and wet-associated conditions. Bacterial and eukaryotic microbial diversity was calculated after rarefaction to minimum sequencing depths of 56,370 reads for bacteria and 51,748 reads for eukaryotes. Boxes show the distribution of repeated observations across two replicate tanks and three sampling days for each treatment. T1:T_Ambient_, T2:T_Dry_, T3:T_Wet_, T4:T_Warm1_, T5:T_Warm-dry1_, T6:T_Warm-wet1_, T7:T_Warm2_, T8:T_Warm-dry2_, T9:T_Warm-wet2_.

**Figure S7 Principal Coordinate Analysis (PCoA) of bacterial and eukaryotic community structures based on Bray-Curtis distance.** Each point represents microbial metrics from a single water sample collected at a specific lake site and date, either during the warm-dry period (2010–2011, *n* = 47) or the warm-wet period (2021–2022, *n* = 45). Differences in overall community structure were evaluated using PERMANOVA with 999 permutations. Marginal boxplots show PC1 and PC2 score distributions, with group differences evaluated by one-way ANOVA followed by Tukey multiple comparisons (α = 0.05).

**Figure S8 Bacterial and eukaryotic community composition at the genus level during warm-dry and warm-wet periods.**

**Figure S9 Redundancy analysis (RDA) of environmental drivers of microbial community structure and functional composition.** (A) RDA ordination showing key environmental factors shaping bacterial community structure during the warm-dry and warm-wet periods, with the lower bar plot showing the percentage of variation explained by significant environmental variables. (B) RDA ordination showing key environmental factors shaping eukaryotic microbial community structure, with the lower bar plot showing the percentage of variation explained by significant environmental variables. (C) RDA ordination showing environmental drivers of KEGG-based functional composition, with the lower bar plot showing the explained variation of significant variables. (D) RDA ordination showing environmental drivers of COG-based functional composition, with the lower bar plot showing the explained variation of significant variables. Orange circles and blue squares represent samples from the warm-dry and warm-wet periods, respectively. Arrows indicate the direction and strength of environmental correlations with microbial community or functional composition. COD: chemical oxygen demand, SSRDS: surface solar radiation downwards sum. Significance levels: **p* < 0.05, ***p* < 0.01, ****p* < 0.001.

**Figure S10 *Z_i_-P_i_* plot categorizing ASVs based on their topological roles within bacterial networks.** Each point represents an ASV. Keystone nodes include module hubs, connectors, and network hubs.

**Figure S11 Topological roles of nodes in bacterial-eukaryotic bipartite networks, identifying module hubs, connectors, and network hubs as keystone nodes.**

**Figure S12** **Functional gene composition during warm-dry and warm-wet periods in field surveys and mesocosm experiments.** (A) PCoA ordination of KEGG-based functional gene composition in field survey samples from the warm-dry and warm-wet periods. (B) PCoA ordination of COG-based functional gene composition in field survey samples. (C) PCoA ordination of KEGG-based functional gene composition in mesocosm samples under simulated future warm-dry and warm-wet scenarios. (D) PCoA ordination of COG-based functional gene composition in mesocosm samples. Differences in overall functional structure were evaluated using PERMANOVA with 999 permutations. Marginal boxplots show PC1 and PC2 score distributions, with group differences evaluated by one-way ANOVA followed by Tukey multiple comparisons (α = 0.05).

**Figure S13** **Microbial functional composition during field surveys and mesocosm experiments.** (A) Relative composition of selected microbial functional pathways in field survey samples from the warm-dry (WD) and warm-wet (WW) periods. (B) Relative composition of selected microbial functional pathways in mesocosm samples under simulated future dry and wet hydrochemical scenarios. The functional categories include methane metabolism, central metabolism, carbon fixation, oxidative phosphorylation, and sulfur metabolism. Stacked bars indicate the relative contributions of subpathways within each functional category.

**Figure S14** **Dominant taxonomic contributors to nitrogen-cycling functions during the warm-dry (WD) and warm-wet (WW) periods.**

**Figure S15 Triangular plots of β-diversity patterns based on Jaccard dissimilarity for eukaryotic microbial communities across warm-dry and warm-wet periods.** Each point is determined by a triplet of values from the Jaccard dissimilarity, Nestedness, and Turnover matrices. The mean values of Jaccard dissimilarity, Nestedness, and Turnover are shown.

# Figure S16 Relative sequence abundance of autotrophic photosynthetic microorganisms during warm-dry and warm-wet periods. The relative abundance of Cyanobacteria is expressed relative to the entire bacterial community, while Chlorophyta, Dinoflagellata, Diatomea, and Ochrophyta are shown relative to the entire eukaryotic microbial community.

# Figure S17 Relative abundance of photosynthesis-related functions during warm-dry and warm-wet periods.

**Figure S18 Light environment during warm-dry and warm-wet periods at Lake Bosten.** SSRDS: surface solar radiation downwards sum; SSRDM: surface solar radiation downwards max; SNSRM: surface net solar radiation max; DURATS: Downward UV radiation at the surface. SSRDS, SSRDM, and SNSRM are displayed as daily values (728 data points in each period), with SSRDS representing the 24 h sum and SSRDM and SNSRM representing daily maxima. DURATS was displayed as monthly total values, with 24 data points in each period. Differences in light environment between the WD and WW periods were assessed using Wilcoxon rank-sum tests. *p* values were adjusted using the Benjamini-Hochberg false discovery rate (FDR) procedure, with *q* < 0.05 considered statistically significant. The ERA5-Land data used in this study are available at https://cds.climate.copernicus.eu/datasets/reanalysis-era5-land?tab=overview. The ERA5 hourly data for downward UV radiation is available at: https://cds.climate.copernicus.eu/datasets/reanalysis-era5-single-levels.

**Figure S19 Functional redundancy was estimated using Tax4Fun2, which predicts functional phylogenetic distributions based on KEGG annotations.** The functional redundancy index (FRI) was computed to indicate the distribution of functions among community members. Differences in FRI between the WD and WW periods were assessed using Wilcoxon rank-sum tests. *p* values were adjusted using the Benjamini-Hochberg false discovery rate (FDR) procedure, with *q* < 0.05 considered statistically significant. WD: warm-dry; WW: warm-wet.

**Figure S20 Long-term changes in population and water consumption in the Kaidu River catchment during 1958−2019.** (A) Population size. (B) Water consumption. Population is expressed as 10^3^ persons, and water consumption is expressed as 10^8^ m^3^.

**Figure S21 Matched site × month sensitivity analyses of microbial diversity, community composition, and environmental shifts between warm-dry and warm-wet periods.** Only Site × Month combinations shared by the warm-dry and warm-wet periods were retained. When multiple samples occurred within the same Type × Site × Month combination, values were averaged before paired comparison. (A–C) Paired comparisons of bacterial Observed ASVs, Shannon diversity and Faith’s PD. (D–F) Paired comparisons of eukaryotic Observed ASVs, Shannon diversity and Faith’s PD. Grey lines connect matched Site × Month pairs. p values were obtained from paired t tests. (G, H) PCoA plots based on Bray-Curtis dissimilarities of ASV relative abundances. Matched blocked PERMANOVA was performed by treating Site × Month pairs as blocking units. (I) Paired effect sizes of key environmental variables. WD, warm-dry; WW, warm-wet; WL, water level; TDS, total dissolved solids; TN, total nitrogen; WT, water temperature; Chl.*a*, chlorophyll-*a*.
